# Supplementary material for: Improved susceptibility weighted imaging at ultra-high field using bipolar multi-echo acquisition and optimized image processing: CLEAR-SWI
Source: Neuroimage. Author manuscript; Available in PMC 2021 Dec 10. (PMC7612087; doi:10.1016/j.neuroimage.2021.118175)
Supplement: Supporting Information [file EMS140488-supplement-Supporting_Information.docx]

Supplementary Information

# Calculation of acquisition time

Tables of repetition time ($T_{R}$), echo times ($T_{E}$) and sampling time per echo ($T_{\mathrm{acq}}$) were calculated for single-echo, monopolar and bipolar multi-echo scans. These tables were used for the calculation of duty cycle per echo and the predicted SNR of the echo-combined magnitude. The excitation time, $T_{\mathrm{exc}}$, and time for gradient spoiling, $T_{\mathrm{spoil}}$, were considered constant, with the values $T_{\mathrm{exc}} = 2.1 \mathrm{ms}$ and $T_{\mathrm{spoil}} = 3.0 \mathrm{ms}$. Tables of ($T_{R}, T_{E}, T_{\mathrm{acq}})$ were created from a range of $T_{\mathrm{acq}}$ values, from which $T_{R}$ and $T_{E}$ were calculated to obtain protocols with maximized sampling time per $T_{R}$. The code for the calculation can be found at

<https://github.com/korbinian90/MriResearchTools.jl/tree/clearswi_publication/src/snr.jl>.

**Single-Echo calculation**

For single-echo, the echo time was calculated as

$$T_{E,\mathrm{SE}} =\frac{T_{\mathrm{EL}}}{2} + T_{\mathrm{exc}}$$

where the required time to acquire one echo, $T_{\mathrm{EL}} = T_{\mathrm{acq}} + 2 T_{\mathrm{ramp}}$ (EL for echo length; assuming no signal sampling on gradient ramps), where $T_{\mathrm{acq}}$ is the sampling time for one echo and $T_{\mathrm{ramp}}$ the time required to ramp the gradient from 0 to $G$, the gradient strength during sampling.

The corresponding $T_{R}$ for single echo is calculated as

$$T_{R,\mathrm{SE}} = T_{\mathrm{exc}} + T_{\mathrm{EL}} + T_{\mathrm{spoil}}$$

$$= T_{E,\mathrm{SE}} +\frac{T_{\mathrm{EL}}}{2}+ T_{\mathrm{spoil}}.$$

**Multi-Echo calculation**

One table ($T_{R}, T_{E}, T_{\mathrm{acq}})$ was generated for each of the number of echoes, $N_{E}$, considered. The bandwidth was set to be the same for all echoes and the echo times were restricted to be multiples of the first echo time.

The spacing between echoes $\Delta T_{E}$ includes the echo length $T_{\mathrm{EL}}$ and for monopolar acquisitions additionally the prewind time $T_{\mathrm{prewind}}$. To have echoes as multiples of the first echo time, $\Delta T_{E}$ must be at least the size of the first echo time. For given $T_{\mathrm{acq}}$, the time of the first echo is calculated according to the single-echo formulation above for $T_{E,\mathrm{SE}}$. Together, the shortest possible spacing between echoes is given as

$$\Delta T_{E} = \max(T_{E,\mathrm{SE}}, T_{\mathrm{EL}} + T_{\mathrm{prewind}})$$

The individual echo times were set to be integer multiples of the echo spacing

$$T_{E,i} = i \Delta T_{E}$$

$$T_{R}=N_{E}\Delta T_{E}+\frac{T_{\mathrm{EL}}}{2}+T_{\mathrm{spoil}}$$

For bipolar acquisitions, $T_{\mathrm{prewind}}=0$ and for monopolar acquisitions $T_{\mathrm{prewind}}$ is calculated in the next part.

To calculate $T_{\mathrm{prewind}}$ for monopolar acquisitions, the gradient moment GM that needs to be reversed is calculated according to

$$\mathrm{GM} = T_{\mathrm{ramp}}\cdot G +\frac{N_{x}}{\gamma\cdot\mathrm{FOV}} ,$$

where $G$ is the gradient strength during sampling, $N_{x}$ the matrix size in the readout direction, $\gamma$ the gyromagnetic ratio, and FOV the field of view in readout direction.

If the ramps played for rewinding do not have a flat top, the maximum gradient strength and the associated time for prewinding is

$$G_{\max}=\sqrt{\mathrm{GM}\cdot R_{\mathrm{slew}}}$$

$$T_{\mathrm{prewind}}=2\sqrt{GM/R_{\mathrm{slew}}},$$

where $R_{\mathrm{slew}}$ is the slew rate of the gradient.

If $G_{\max}$ is bigger than the hardware gradient limit $G_{\mathrm{limit}}$, the following calculation with a flat region on top is used for $T_{\mathrm{prewind}}$:

$$T_{\mathrm{ramp}}=G_{\mathrm{limit}}/R_{\mathrm{slew}}$$

$$\mathrm{GM}_{\mathrm{ramp}}=T_{\mathrm{ramp}}\cdot G_{\mathrm{limit}}$$

$$\mathrm{GM}_{\mathrm{box}}=GM-\mathrm{GM}_{\mathrm{ramp}}$$

$$T_{\mathrm{box}}=\mathrm{GM}_{\mathrm{box}}/G_{\mathrm{limit}}$$

$$T_{\mathrm{prewind}}=2T_{\mathrm{ramp}}+T_{\mathrm{box}}$$

**Duty Cycle**

The duty cycle for a multi-echo sequence with a given echo spacing $\Delta T_{E}$ is calculated as

$$D = T_{\mathrm{sample}} / \Delta T_{E},$$

which is

$$D_{\mathrm{bip}} =\frac{\Delta T_{E} - 2T_{\mathrm{ramp}}}{\Delta T_{E}}$$

$$= 1 -\frac{2T_{\mathrm{ramp}}}{\Delta T_{E}}$$

and

$$D_{\mathrm{mono}} =\frac{\Delta T_{E} - 2T_{\mathrm{ramp}} - T_{\mathrm{prewind}}}{\Delta T_{E}}$$

$$= 1 -\frac{2T_{\mathrm{ramp}} + T_{\mathrm{prewind}}}{\Delta T_{E}} .$$

# Comparison of Standard SWI reference from Multi-Echo Data and Single-Echo Data

In the patient study, we simulated the images which would have been generated with a single-echo scan from acquired multi-echo data. This excluded the possibility that patient compliance (particularly motion) might be different between single-echo and multi-echo acquisitions (which would preclude a fair comparison), and reduced the scan duration for the patients. Here, we demonstrate, in a healthy subject for whom both single-echo (*NE1*) and multi-echo (*NE6*) were available, that the simulated NE1 images do accurately represent the data that would have been acquired with a single-echo scan. As can be seen from Figure B‑1, the contrast and signal dropouts of the simulated magnitude are very similar to the single-echo scan. Also, after homodyne filtering, the simulated phase closely corresponds to the acquired phase, with similar artefacts. The resulting SWI image quality is comparable to the acquired scan and presents similar artefacts in the same places, validating the comparison in the patient study.


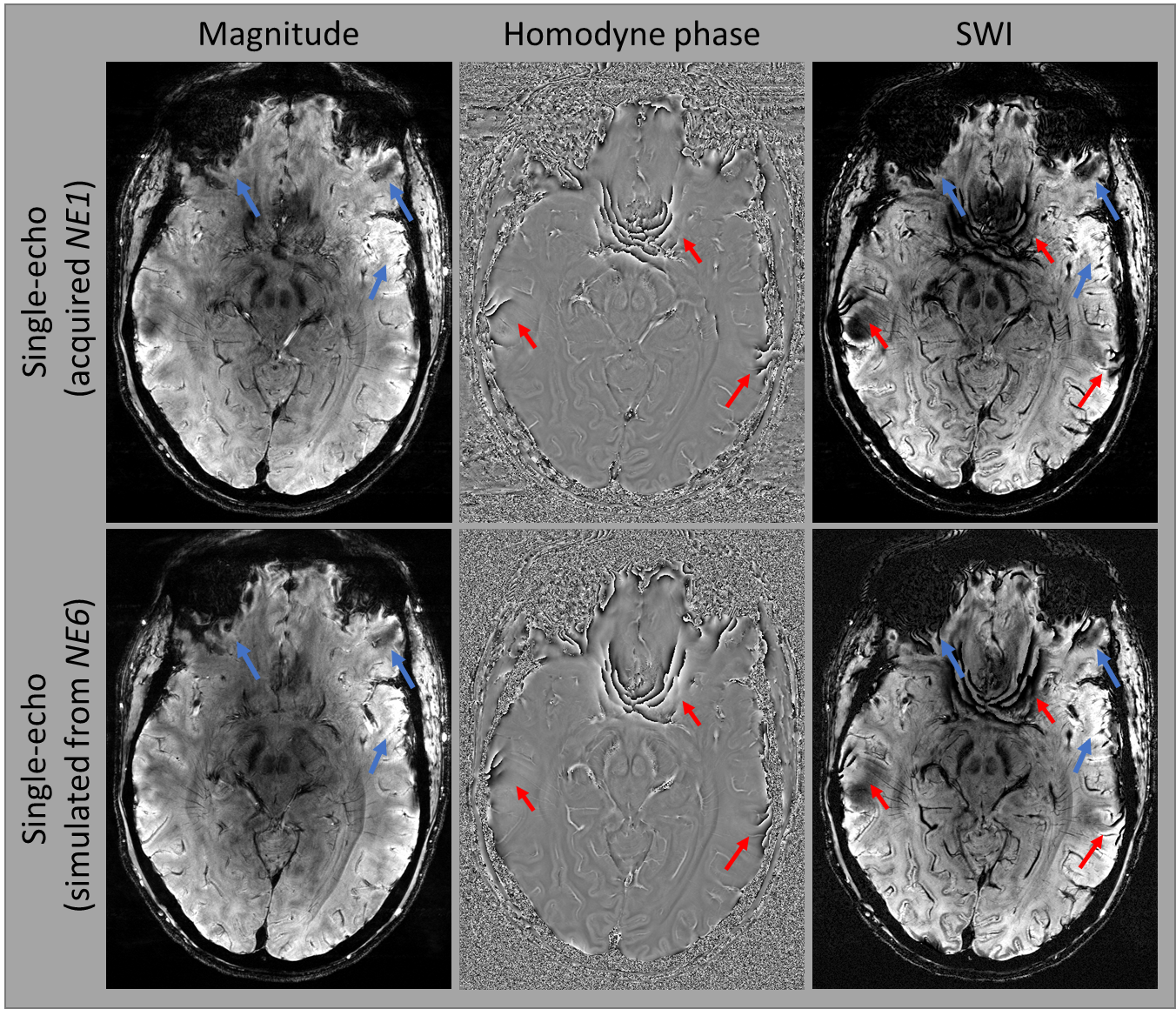


Figure B‑1: Comparison of acquired single-echo data (NE1) and simulated single-echo data derived from the NE6 scan. The magnitude contrast is very similar and the blue arrows point to equally strong signal loss and inhomogeneity artifacts in the magnitude. The red arrows point to similar phase artifacts in the homodyne filtered phases (homodyne phase exported from scanner for the scan NE1). Minor differences are attributable to flow artefacts and slightly different slice positioning (motion between the acquisitions). The resulting SWI has some differences in the exact location of artefacts but contains artifacts of similar severity in the same regions.

# Phase Masking

The following comparison of the phase masking function was performed on the same multi-echo dataset as is presented in the main manuscript (Table 1). For comparison of the different phase masking algorithms, the coil-combined phase was Laplacian unwrapped and echoes were combined and high-pass filtered identically to the procedure in the CLEAR-SWI pipeline. The processed phase image after high-pass filtering was used to compare the different SWI phase masking functions.

The images in Figure C‑1 show phase mask values directly after application of the specific phase masking function. The top left image corresponds to linear phase masking, which is very weak. Stronger phase masking is achieved, as is typical in Standard SWI, with more multiplications, which is shown in the top row of images, but noise is amplified. The bottom row shows phase masking with the proposed CLEAR-SWI sigmoidal function with different values of the adjustable Level parameter. The Level parameter increases from left to right, generating stronger phase weighting. CLEAR-SWI phase masking is less noisy than Standard SWI masking with similar contrast.

A level parameter of 4 (second image from left, bottom row) was chosen to achieve the closest correspondence to the contrast generated by the vendor’s Standard SWI implementation (Siemens 7T MAGNETOM VB17 implementation of SWI).


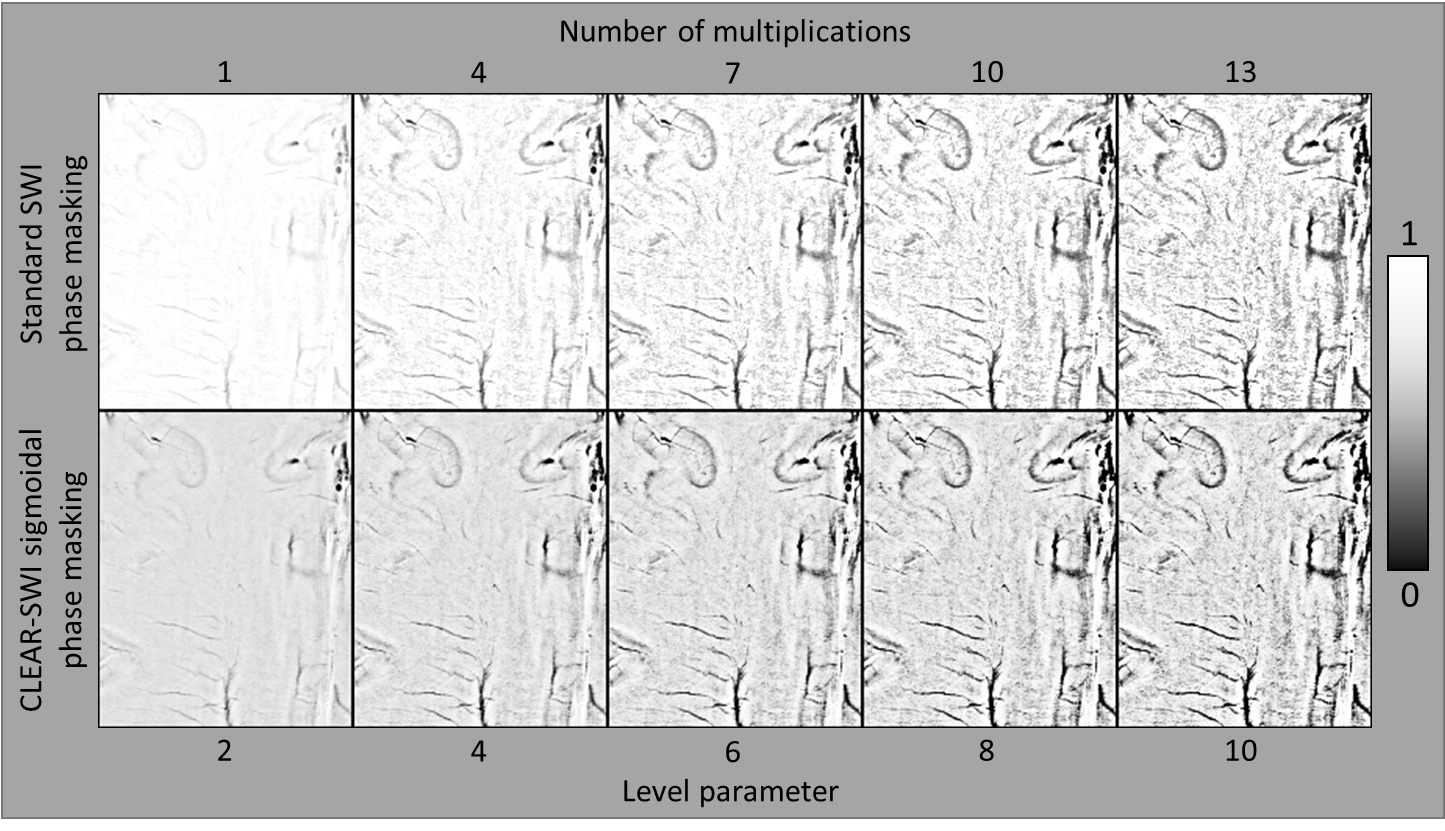


Figure C‑1: Comparison of Standard SWI phase masking (top row) with sigmoidal phase masking (bottom row) used in CLEAR-SWI. The images are arranged to show similar contrasts for vertically aligned images. Background noise becomes amplified for higher multiplication factors in Standard SWI phase masking. With sigmoidal masking, increasing the level parameter (defined in Equation 12 in the main manuscript) only slightly increases the background noise, but allows fine features to still be distinguished from noise.

**Comparison of 2D and 3D filtering and different σ sizes**

The comparison of different high-pass filtering settings was performed with the standard CLEAR-SWI pipeline on the multi-echo dataset from the main manuscript. The high-pass filtering parameter σ was varied and the processed SWI phase is shown after phase masking. For this comparison, the sigmoidal phase masking function was set to $level=4$.

The high-pass filter subtracts a Gaussian low-pass filtered image from the input. The values used in the 2D Gaussian filters were $\sigma= [1, 2, 3, 4, 5, 8, 12, 20]$ voxels in the x- and y-direction. The 3D Gaussian filter used the same sizes in the x- and y-direction, and additionally $\sigma_{z}=\sigma_{x,y}/4$ in the z-direction (due to the voxel aspect ratio of 4:1).

Figure C‑2 presents 2D and 3D smoothing results with increasing σ values from left to right. The background noise is higher with smaller σ and only fine structures and borders are apparent, rather than regions. For large values of σ, noise is reduced and larger regions gain contrast, but some fine features (such as small vessels) have reduced visibility.


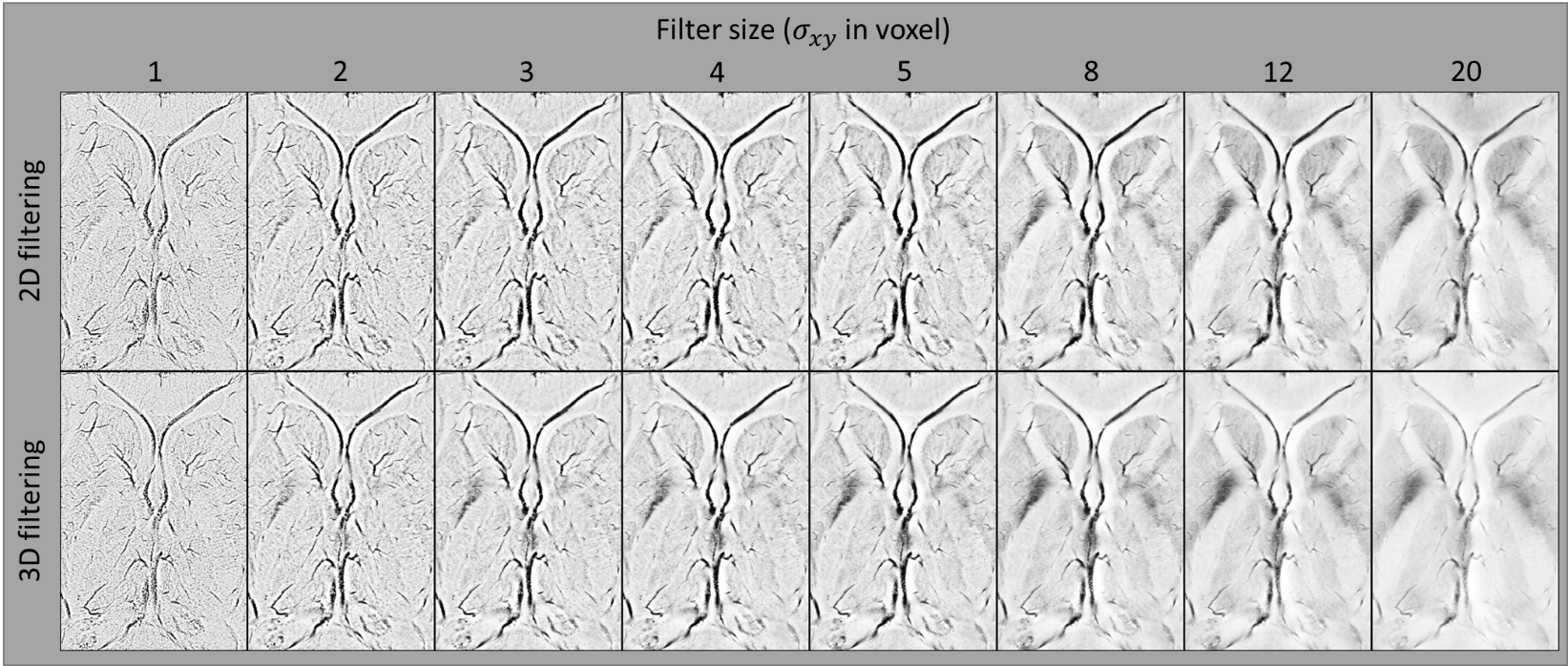


Figure C‑2: Comparison of different σ values for 2D and 3D Gaussian high-pass filtering. The processed phase after phase masking is shown. 2D smoothing was applied in the top row and 3D smoothing in the bottom row, where $\sigma_{z}=\sigma_{xy}/4$. Larger smoothing kernels can visualize differing susceptibility distributions for different tissue types, however some fine detail is lost. 3D filtering tends to be less noisy, but also very fine structures are less visible.

Figure C‑3 shows a problematic region for phase filtering, due to strong background ΔB_0_ variations. For large filter sizes this leads to strong artefacts and large σ sizes also eliminate detail close to boundaries.


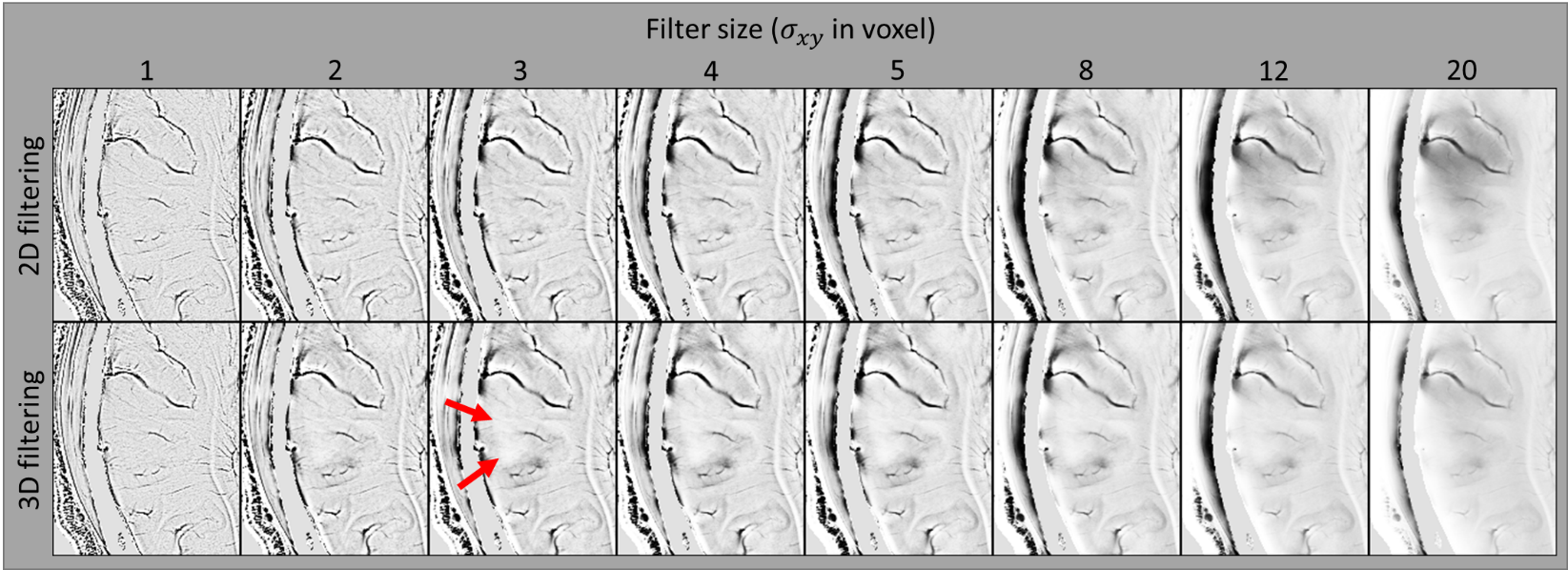


Figure C‑3: Comparison of filtering outcomes with different σ values for 2D and 3D Gaussian high-pass filtering. The processed phase after phase masking is shown. The section shown is an enlargement of a region with large ΔB_0_ variation, which causes severe problems for larger filter sizes. 3D filtering has inhomogeneity problems even for low filter sizes (red arrows), where a whole area has reduced visibility of fine structures.

The results of 3D smoothing are presented in the bottom rows of Figure C‑2 and Figure C‑3. 3D smoothing has reduced noise compared to 2D smoothing but introduces artefacts due to the large voxel size in the z-direction. This can be seen especially in Figure C‑3 with $\sigma=3$, where already some parts are very bright with most details not visible in these regions.

As a robust default, 2D smoothing with $\sigma=4$ (top row, fourth image from left) was chosen. This shows all the detail and the effect of artefacts is very limited and has the advantage that processing can be performed on a slice-by-slice basis.

In general, the output phase mask is weaker with increasing σ, which can be counteracted by choosing a higher level parameter of the phase masking function. 3D smoothing would be expected to perform better for isotropic acquisitions.

# Comparison of contrast with different magnitude multi-echo combination weightings

In SNR-weighted CLEAR-SWI, the signal intensity of certain areas (e.g. basal ganglia, Figure D‑1), is not as low as in Standard SWI. This contrast difference originates in the magnitude, which is i) less subject to T2* decay due to the echo weighting used in this case, which was to achieve maximum SNR and ii) because of B1- inhomogeneity, which causes the image to be darker in the center. A higher tissue contrast (i.e. lower signal in the basal ganglia, and other regions with short T2*) can be achieved in CLEAR-SWI using CNR-weighted combination at the cost of slightly increased signal dropouts. An additional possibility to increase contrast is to apply the softplus scaling function (Section 3.10.3 in the main manuscript). The parameters of this were chosen to result in similar contrast to Standard SWI. The basal ganglia structures in Figure D‑1 appear very dark in Standard SWI, which is mainly due to uncorrected inhomogeneity.


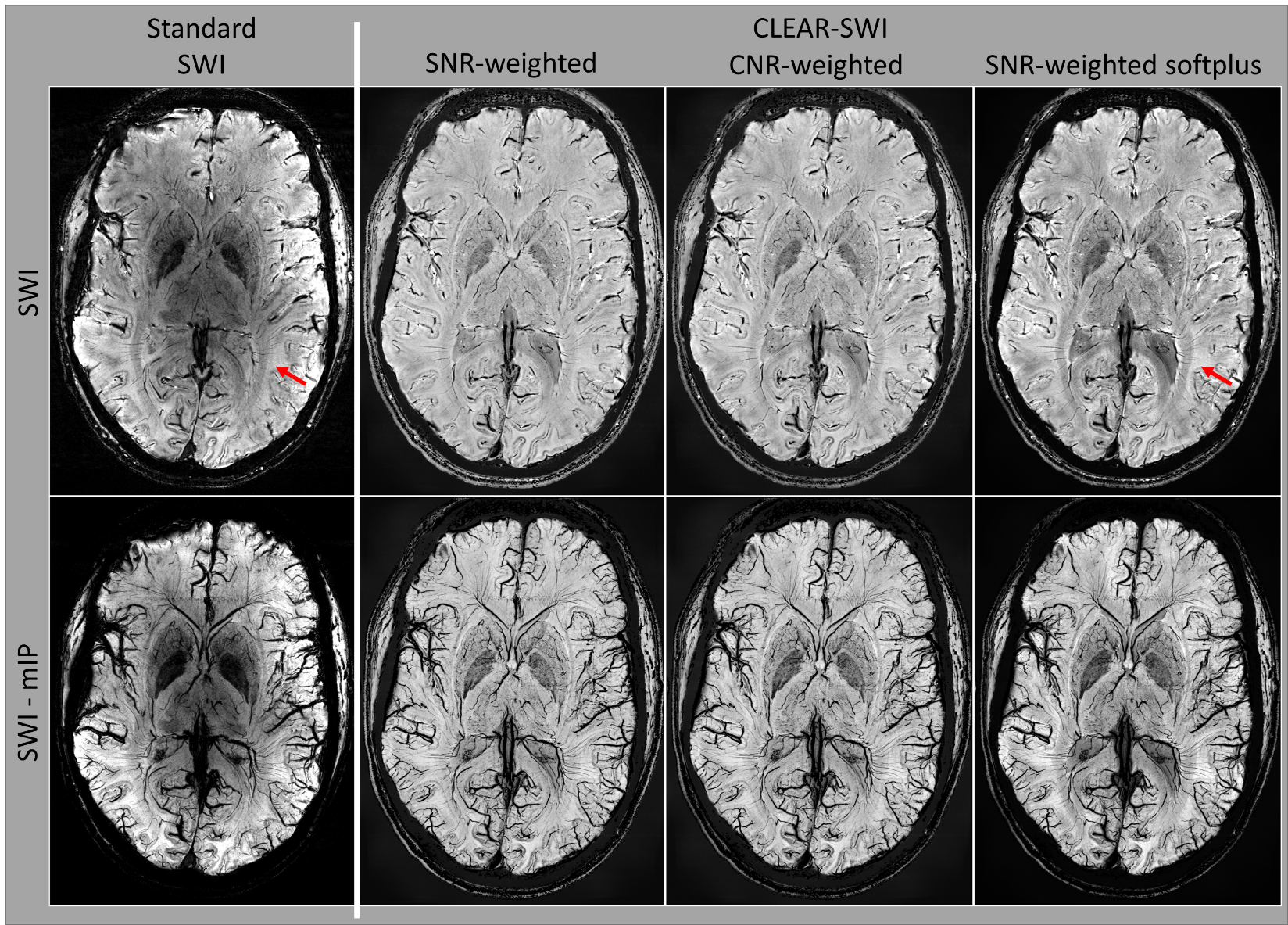


Figure D‑1: Contrast comparison between Standard SWI and CLEAR-SWI with different settings: The contrast in the basal ganglia is higher in Standard SWI than in SNR-weighted CLEAR-SWI (e.g. the putamen and pallidum appear darker). With CNR-weighting (gm/wm), the contrast is slightly increased. The contrast can be further increased using the softplus scaling function, here applied to the SNR-weighted magnitude (Section 3.10.3 in the main manuscript). The very dark appearance of the basal ganglia in Standard SWI stems from the not-corrected homogeneity artefact. The arrow points to the optic radiation, which is difficult to see in Standard SWI, but has high contrast in CLEAR-SWI.

# Comparison of CLEAR-SWI with other multi-echo SWI methods

The SWI method from Quinn et al. (2014) , which was developed for SWI at 3 T, was performed according to the publication other than that the homodyne filter size was reduced from 30% of the FOV to 3T/7T * 30% FOV = 13% FOV. The filter size was reduced to adjust for the stronger expected background field, which, however, only slightly mitigated the homodyne filtering artefacts.

GEPCI was implemented as described in the publication (Luo et al., 2012). Complex fitting was performed by splitting the complex data into real and imaginary parts and fitting a vector of twice the size of the number of echoes (this was confirmed by the authors of GEPCI to be method they used, with Matlab lsqcurvefit). This was found not to be robust for the data in our study. For voxels with larger phase values / higher frequencies, the fitting converged on values which – judged by the fact that they differed dramatically from neighboring voxels - were wrong (probably a local minimum rather than a global minimum). The same problem was observed with fitting in Julia (using Levenberq-Marquadt) and Matlab (lsqcurvefit, trust-region-reflective and Levenberg-Marquadt) and was only worsened with bounded fitting. Though there might be solutions to this problem it has not been further investigated, and regions with these fitting problems were avoided in the SNR and CNR comparisons.

To compare the CNR from the different SWI methods, Wilcoxon sign rank tests were performed between all pairs of methods for each tissue type. The p values shown in Table E-1 correspond to Figure 7 of the main manuscript, which shows the mean CNR achieved with each method. Each sample was included in 4 comparisons. To correct for multiple testing, the p value at which the findings are significant was reduced to $p=0.05/4$ (Bonferroni correction). The total number of ROI pairs were 261, and the total number per tissue type was 87 (GM), 48 (GP), 64 (OR), 26 (RN), 36 (SN) and the total number of ROI pairs per volunteer was 55, 46, 53, 53, and 54.

Table E-1: P values obtained using the Wilcoxen signed rank test matching Figure 7 from the main manuscript. The arrows point to the method with significantly higher CNR. Because of multiple testing of the same data (n=4), the Bonferroni corrected significance level is at p = 0.05 / 4. All entries which were not significant are marked “n.s.”. The CNR values for GEPCI have larger fluctuations and therefore some entries of GEPCI are not significant.

| Region | Method | CLEAR-SNRw | CLEAR-CNRw | Quinn | GEPCI |
| --- | --- | --- | --- | --- | --- |
| Gray Matter | **CLEAR-CNRw** | 0.010 🡱 |  |  |  |
|  | **Quinn** | 0.094 **n.s.** | <0.001 🡰 |  |  |
|  | **GEPCI** | <0.001 🡱 | <0.001 🡱 | <0.001 🡱 |  |
|  | **Single echo** | <0.001 🡱 | <0.001 🡱 | <0.001 🡱 | 0.502 **n.s.** |
| Globus Pallidus | **CLEAR-CNRw** | <0.001 🡰 |  |  |  |
|  | **Quinn** | <0.001 🡰 | <0.001 🡱 |  |  |
|  | **GEPCI** | <0.001 🡰 | 0.151 **n.s.** | 0.780 **n.s.** |  |
|  | **Single echo** | 0.002 🡱 | <0.001 🡱 | <0.001 🡱 | <0.001 🡱 |
| Optic Radiation | **CLEAR-CNRw** | <0.001 🡱 |  |  |  |
|  | **Quinn** | 0.031 **n.s.** | <0.001 🡰 |  |  |
|  | **GEPCI** | <0.001 🡱 | <0.001 🡱 | <0.001 🡱 |  |
|  | **Single echo** | <0.001 🡱 | <0.001 🡱 | <0.001 🡱 | 0.902 **n.s.** |
| Red Nucleus | **CLEAR-CNRw** | <0.001 🡰 |  |  |  |
|  | **Quinn** | <0.001 🡰 | <0.001 🡱 |  |  |
|  | **GEPCI** | 0.003 🡰 | 0.075 **n.s.** | 0.532 **n.s.** |  |
|  | **Single echo** | <0.001 🡱 | <0.001 🡱 | <0.001 🡱 | <0.001 🡱 |
| Substantia Nigra | **CLEAR-CNRw** | <0.001 🡰 |  |  |  |
|  | **Quinn** | <0.001 🡰 | <0.001 🡱 |  |  |
|  | **GEPCI** | 0.010 🡰 | <0.001 🡱 | 0.033 **n.s.** |  |
|  | **Single echo** | 0.002 🡱 | <0.001 🡱 | <0.001 🡱 | <0.001 🡱 |

Figure E‑1 presents the magnitude contrast that is obtained with different multi-echo combination methods and with single-echo. CLEAR SNR-weighted has the weakest contrast, Quinn et al. (averaging) and CLEAR CNR-weighting have slightly more contrast, CLEAR SNR-weighting with softplus scaling is similar in contrast strength to single-echo but has reduced signal dropouts. GEPCI has a contrast very closely resembling single-echo but has an equal or slightly stronger signal dropouts than single-echo. By visual inspection, the noise level appears higher in both GEPCI and single-echo as compared to the other magnitude images.


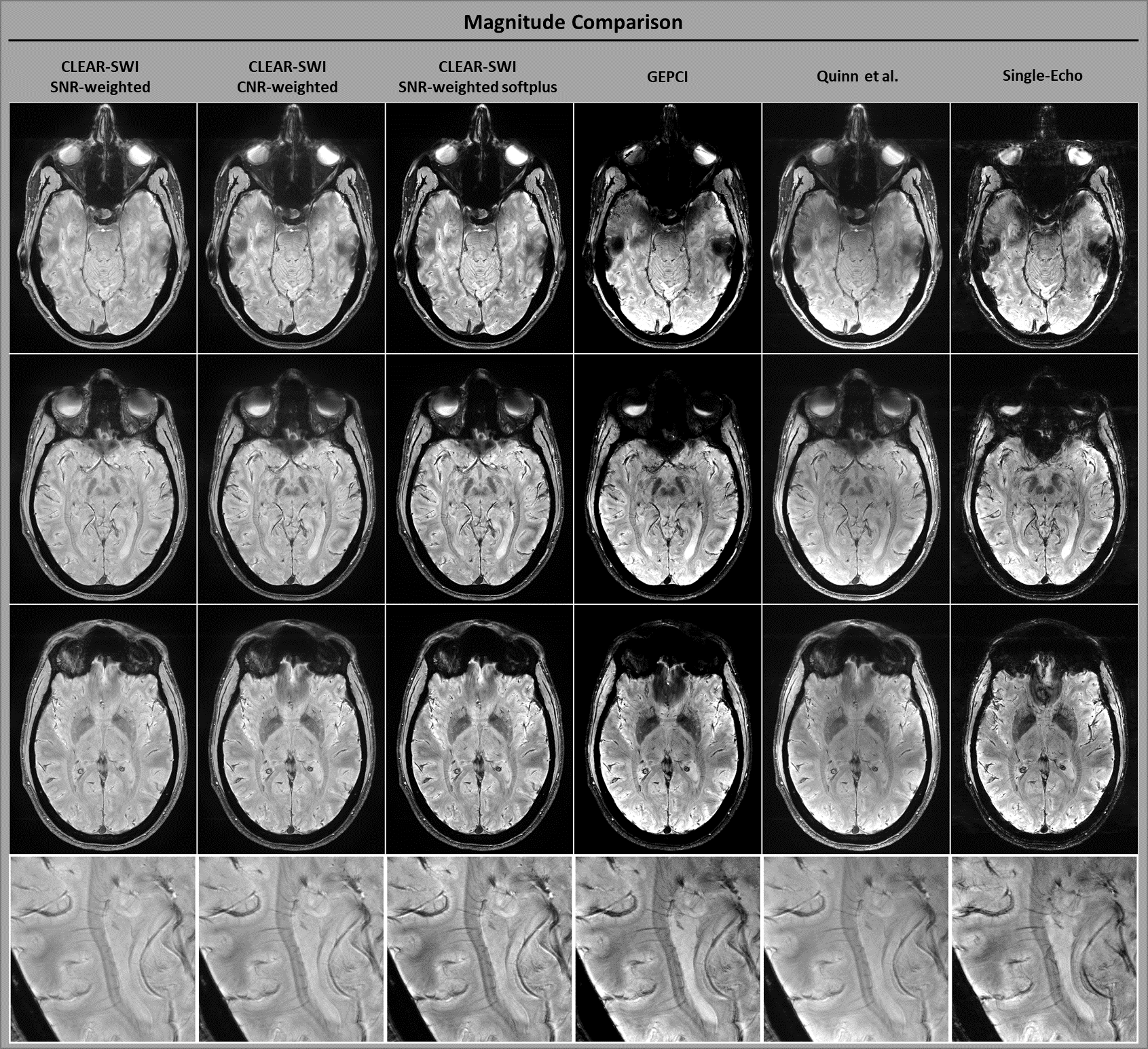


Figure E‑1: Visual comparison of the contrast in the magnitude. 3 different slices and one zoom-in region are shown. The shown images are the respective magnitude images and no phase weighting has been performed. GEPCI and single-echo have strong signal dropouts in near the ear canals, in frontal regions and the eyes, where the other multi-echo combination methods still recover the signal in large parts.

Figure E‑2 compares the visibility of veins with different magnitude combination settings in CLEAR-SWI and the other multi-echo SWI methods GEPCI and Quinn et al. The first example shows a case in which the Quinn et al. SWI had severe artefacts from phase processing but in which the central vein complex is not affected. Comparing the veins, they are best visualized in CLEAR-SWI, slightly less well resolved in Quinn et al. and hard to distinguish from tissue in GEPCI (red arrow). The second example was chosen because it was free of artefacts in GEPCI and Quinn et al.. While most veins are visible in all methods, they are most clearly visualized in CLEAR-SWI.


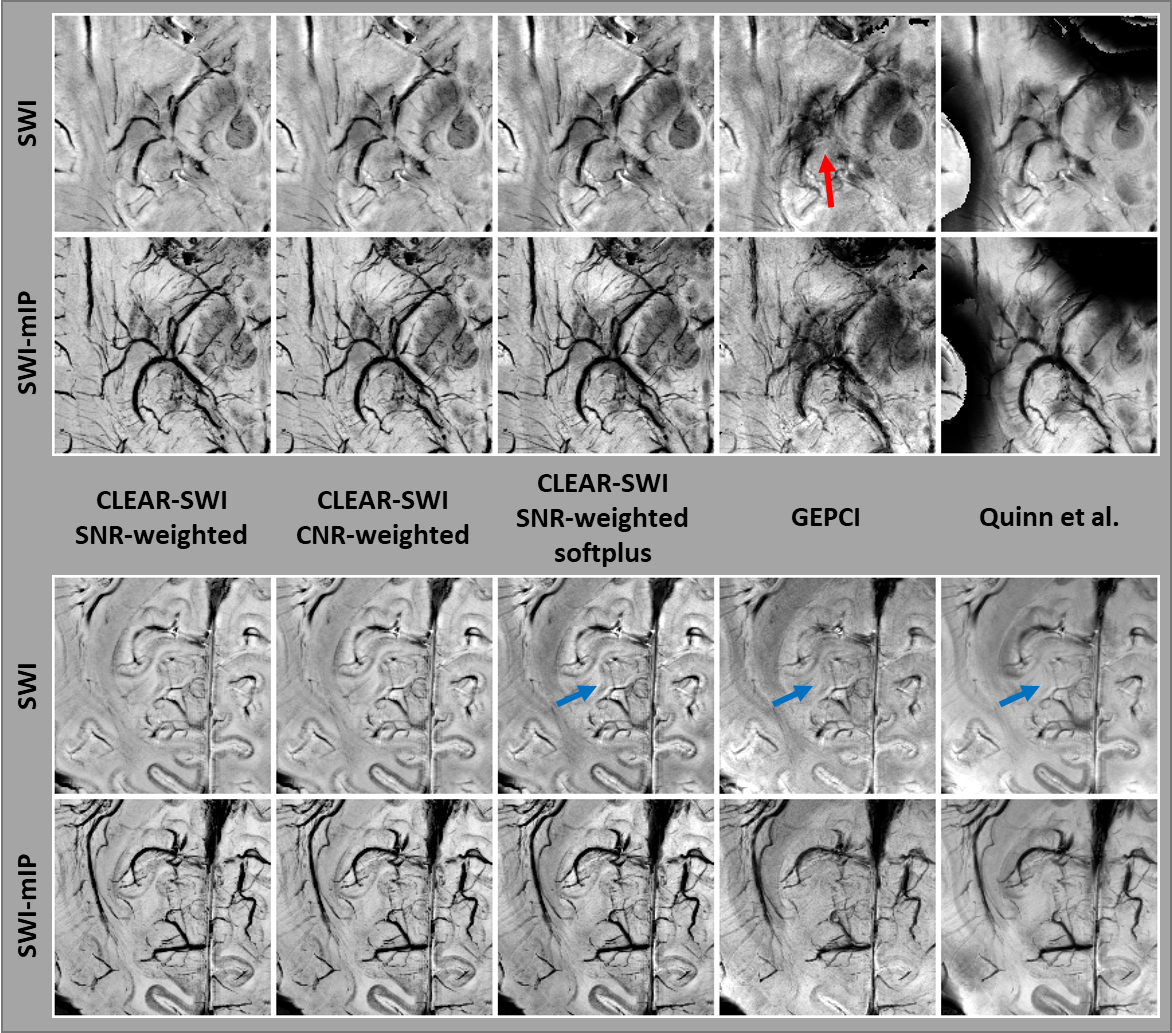


Figure E‑2:Comparison of the visibility of veins between the multi-echo SWI methods in two regions. For each region, the first row is SWI and the second row minimum intensity projection SWI. The red arrow points to an area in which it is difficult to detect the veins in GEPCI, while the other methods produce clearly visible veins. The Quinn et al. SWI of the first region is strongly affected by phase artefacts on the edges of the presented region. In the second regions, the blue arrows point to a region with small veins which are best visualized in CLEAR-SWI and are most difficult to observe in SWI from Quinn et al. In the SWI-mIP images, most veins are slightly better distinguishable in CLEAR-SWI. There is no substantial difference between the different CLEAR-SWI magnitude combination methods (CLEAR-SWI SNR-weighted and CLEAR-SWI CNR-weighted).

Figure E‑3 shows a general comparison of SWI and SWI-mIP results of CLEAR-SWI, Quinn et al. and GEPCI, as well as presents artefacts that were encountered in Quinn et al. and GEPCI. The top row shows an axial slice around the top of the ventricles. The general appearance is good in all three SWI methods, but CLEAR-SNR is more homogeneous and has less signal loss at the front of the brain. The lower part of the figure shows a detail view of a slice at the level of the putamen. The SWI from Quinn et al. is highly corrupted in this area by phase artefacts. The GEPCI SWI is generally of high quality, but structures and veins are still more distinct in CLEAR-SWI. In the GEPCI SWI-mIP, errors from the unstable fitting algorithm are visible. These problems probably occur due to assuming a wrong minimum in complex fitting and only occur for certain scans. They could possibly be resolved with better adapted fitting algorithms, but point to the fragility of this step, nonetheless.


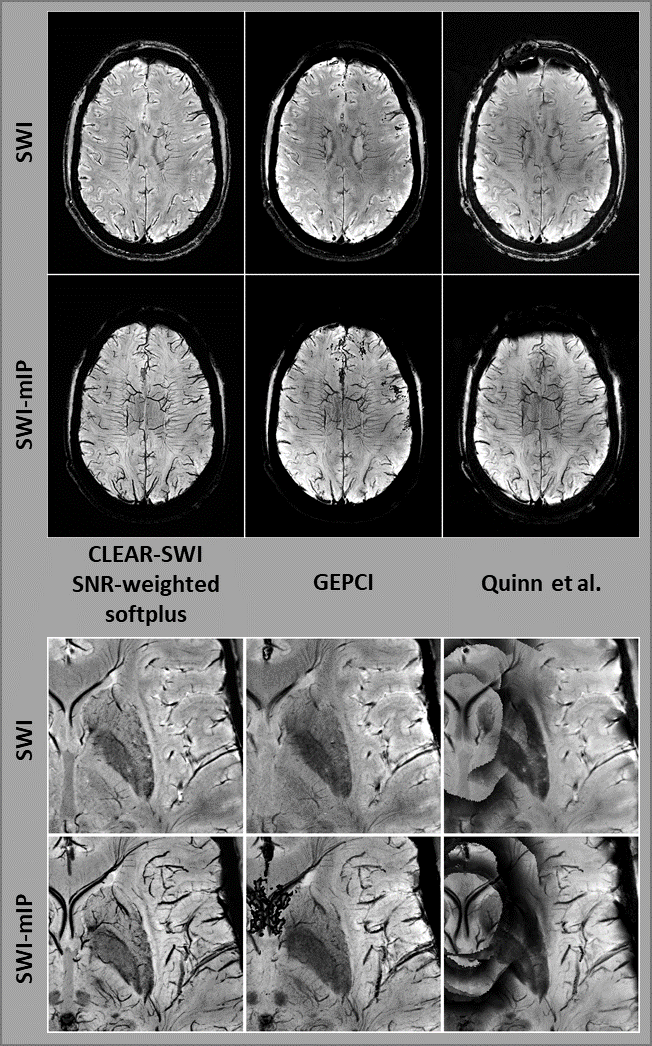


Figure E‑3: SWI Comparison between CLEAR-SWI, GEPCI and Quinn et al. The top rows show that CLEAR-SWI has a more homogeneous appearance and a high visibility of veins both in the SWI and SWI-miP. The bottom row shows strong image arfetacts that occur in Quinn et al. even for a central brain region, which could be even mistaken for veins (see putamen). A number of fitting errors are visible In the GEPCI SWI-mIP.

# Application of CLEAR-SWI steps to Single-Echo Data

Individual steps of CLEAR-SWI were applied to single-echo data: for magnitude processing, the homogeneity correction and non-linear scaling and for phase processing, the Laplacian unwrapping, phase filtering and sigmoidal scaling.

ASPIRE is used for coil combination in multi-echo CLEAR-SWI. In applying CLEAR-SWI steps to single-echo data, an alternative coil combination method is required. The standard homodyne filtering that is used for single-echo coil combination leads to homodyne wrap artefacts and other coil combinations like adaptive combine (as implemented at Siemens 7 T MAGNETOM) lead to open-ended fringe line artefacts. At most systems with 3 T or lower field strength, the phase combination is no problem and high-quality combined phase can be obtained with vendor reconstruction. At 7 T, possibilities include COMPOSER (Robinson et al., 2017b) (although that needs a short reference scan) and the virtual receiver coil (Parker et al., 2014; Robinson et al., 2017a). To avoid the phase combination problems which can affect single-echo combination methods and allow a comparison with multi-echo CLEAR-SWI, the assessment here was performed on simulated single-echo data using the multi-echo acquisition *NE6 SNR* that was combined using ASPIRE*.* The single-echo simulation was performed with a simulated echo time of 20 ms as described in the main manuscript *3.5 Standard SWI reference from multi-echo data* and *Supplementary Information, Appendix B*. Both, Standard SWI and CLEAR-SWI steps were applied to the identical simulated single-echo data.

The parameters were all chosen to be identical to the parameters used for multi-echo CLEAR-SWI, except for magnitude non-linear scaling. As the single-echo contrast is already quite strong, non-linear scaling can be avoided or performed with a parameter which generates less magnitude contrast. Here, the cutoff parameter *b* was reduced to 0.2 times the 0.8-quantile (from 0.5 times the 0.8-quantile used in multi-echo CLEAR-SWI). The CLEAR-SWI steps were applied to single-echo data using the same Julia program^[[1]](#footnote-2)^ as was used for multi-echo CLEAR-SWI.

The images presented in this section were automatically scaled into the [0;1] grayscale range via

Image = Image / quantile(Image, 0.8) * 0.75.

Figure F‑1 shows a lower slice, where Standard SWI had strong homodyne artefacts in the frontal area. With the CLEAR-SWI phase processing steps, no homodyne artefacts were present, but still a large area was corrupted due to signal loss. In multi-echo CLEAR-SWI, the signal loss in the frontal area is greatly reduced and only slightly visible. The inhomogeneity of the magnitude visible in Standard SWI was also corrected with CLEAR-SWI steps. The calculation of a minimum intensity projection (mIP) also benefitted from the increased homogeneity and removed homodyne artefacts.


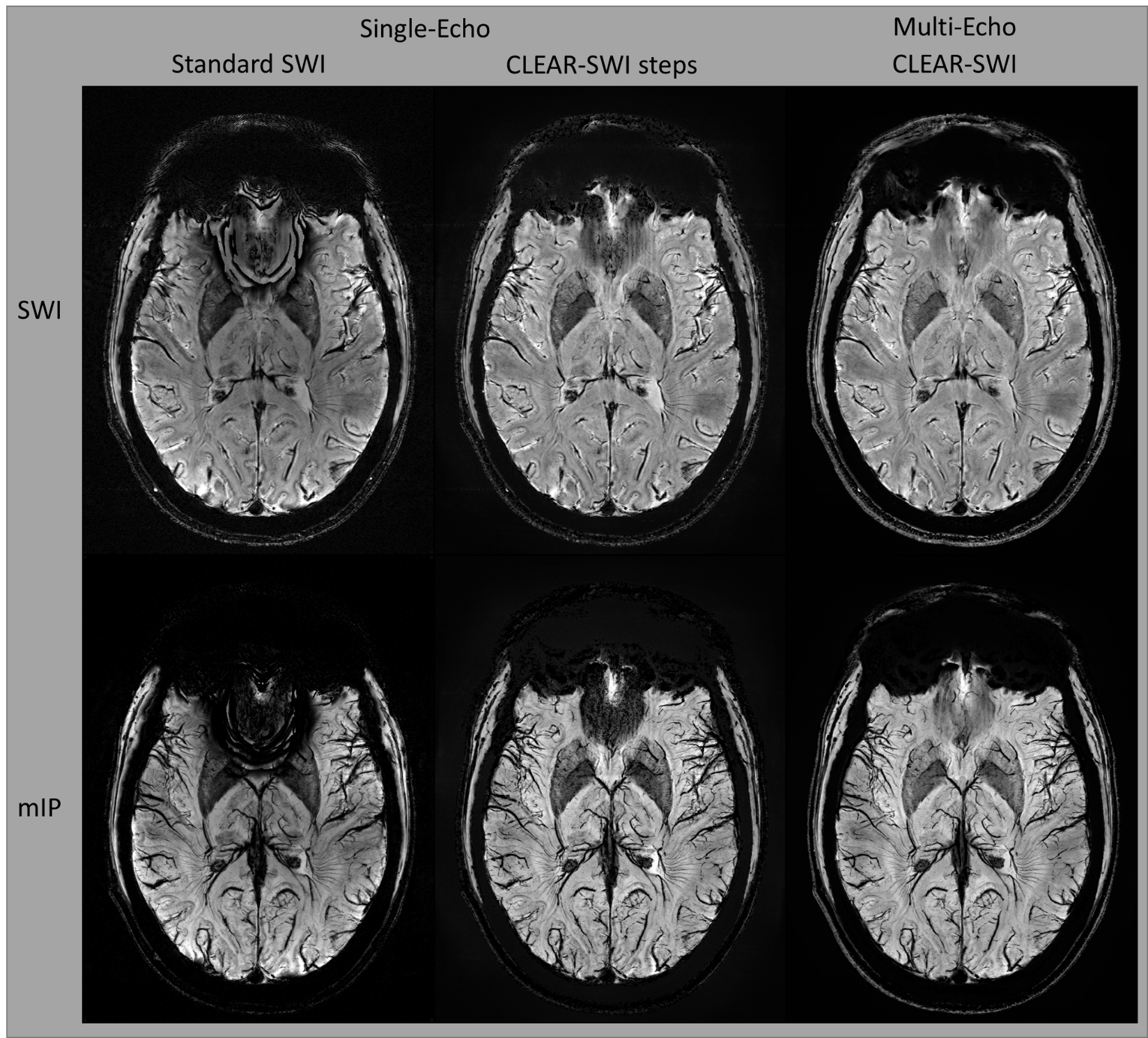


Figure F‑1: Comparison of Standard SWI and CLEAR-SWI applied on simulated single-echo data. SWI and minimum intensity projections are shown. Standard SWI has strong homodyne artefacts in the frontal region and increased intensity towards the edges of the brain, especially dorsally. Both artefacts are not present in CLEAR-SWI. The tissue contrast in the multi-echo CLEAR-SWI is slightly weaker than in the simulated single-echo variant, however, the contrast strength can be adjusted in the non-linear scaling.

# Comparison of Standard SWI and CLEAR-SWI in all 5 volunteers

Standard SWI (*NE1*) is compared with CLEAR-SWI (*NE6*) for a single subject in figures 9 and 10 in the main manuscript. In this section, we show results for all subjects and with two variants of Standard SWI; *NE1* (same as in main manuscript; in which signal was acquired for the whole available acquisition window, with a bandwidth of 60 Hz/pixel) and *Sup NE1* (in which a more typical bandwidth of 100 Hz/pixel and a slightly later echo time of 22.5 ms was used). SNR-weighted magnitude combination was used for CLEAR-SWI with softplus scaling. In Figure G‑1, consistent with the single-subject results in the main manuscript, more, finer veins are resolved in CLEAR-SWI, and images are less noisy and more homogeneous. All images presented in this section were automatically scaled into the [0;1] grayscale range via

Image = Image / quantile(Image, 0.8) * 0.75.

This scaling provided better results for inhomogeneous data (brightening artefact in Standard SWI) than simple rescaling via maximum and minimum into the range [0;1].


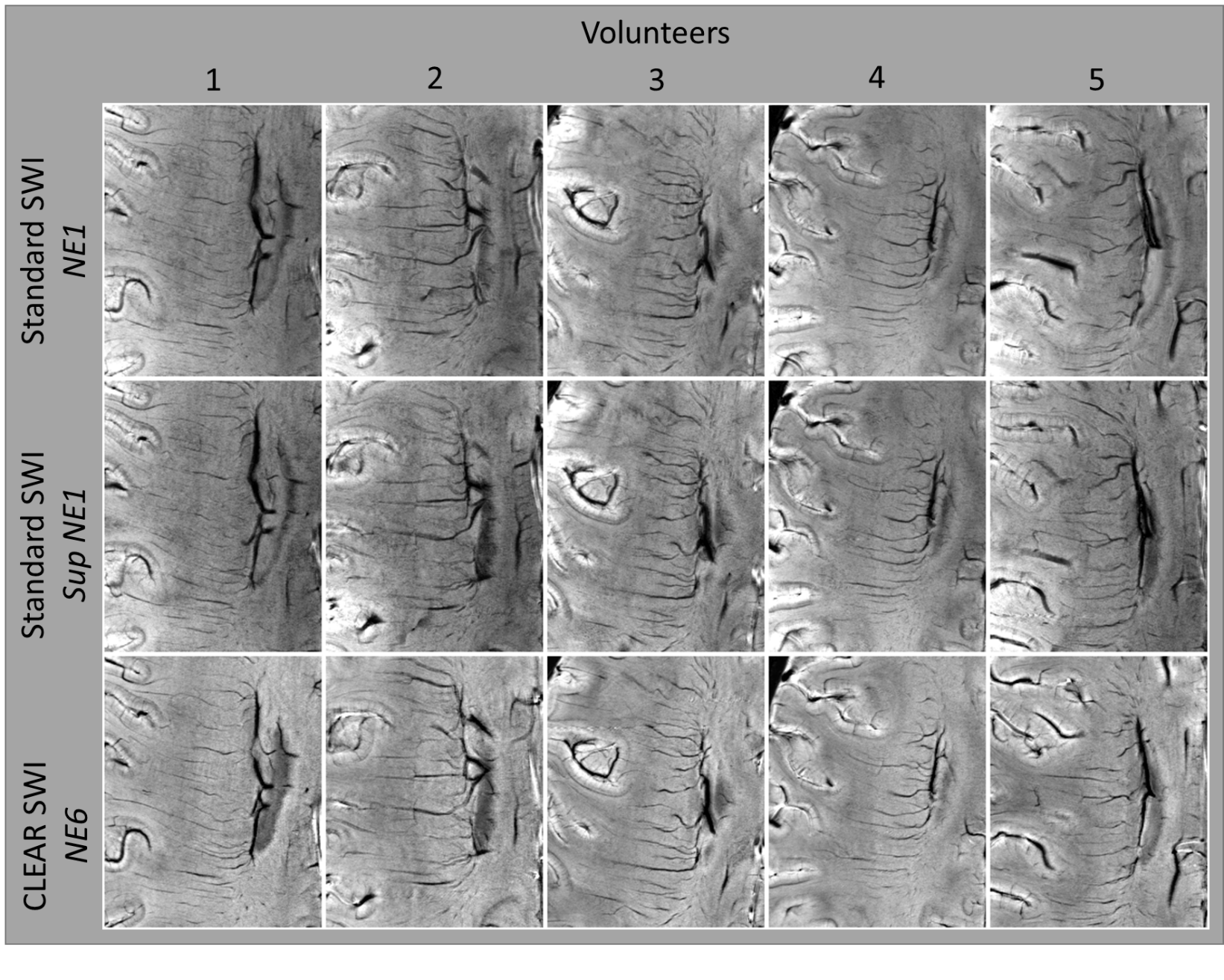


Figure G‑1: Comparison of Standard SWI (bandwidth in top row 60 Hz/px; mid row 100 Hz/px) with CLEAR-SWI (SNR-weighted softplus). The contrast between veins and surrounding tissue is considerably increased in CLEAR-SWI. Note that the best matching slices were selected for comparison, but that slice positions not identical between acquisitions due to slight subject motion.

In Figure G‑2, Figure G‑3 and Figure G‑4, we extend the single-subject results of Figure 9 in the main manuscript by showing results for all subjects and both Standard SWI variants (*NE1* and *Sup NE1*) at three levels – a central slice, at circa MNI152 z=+15 (Figure G‑2), a mid-ventral slice at circa MNI152 z=-7 (Figure G‑3) and a ventral slice at circa MNI152 z=-15 (Figure G‑4). These confirm the four main advantages of CLEAR-SWI over Standard SWI i) reduced signal loss ii) an absence of phase artefacts iii) increased CNR and iv) improved homogeneity.


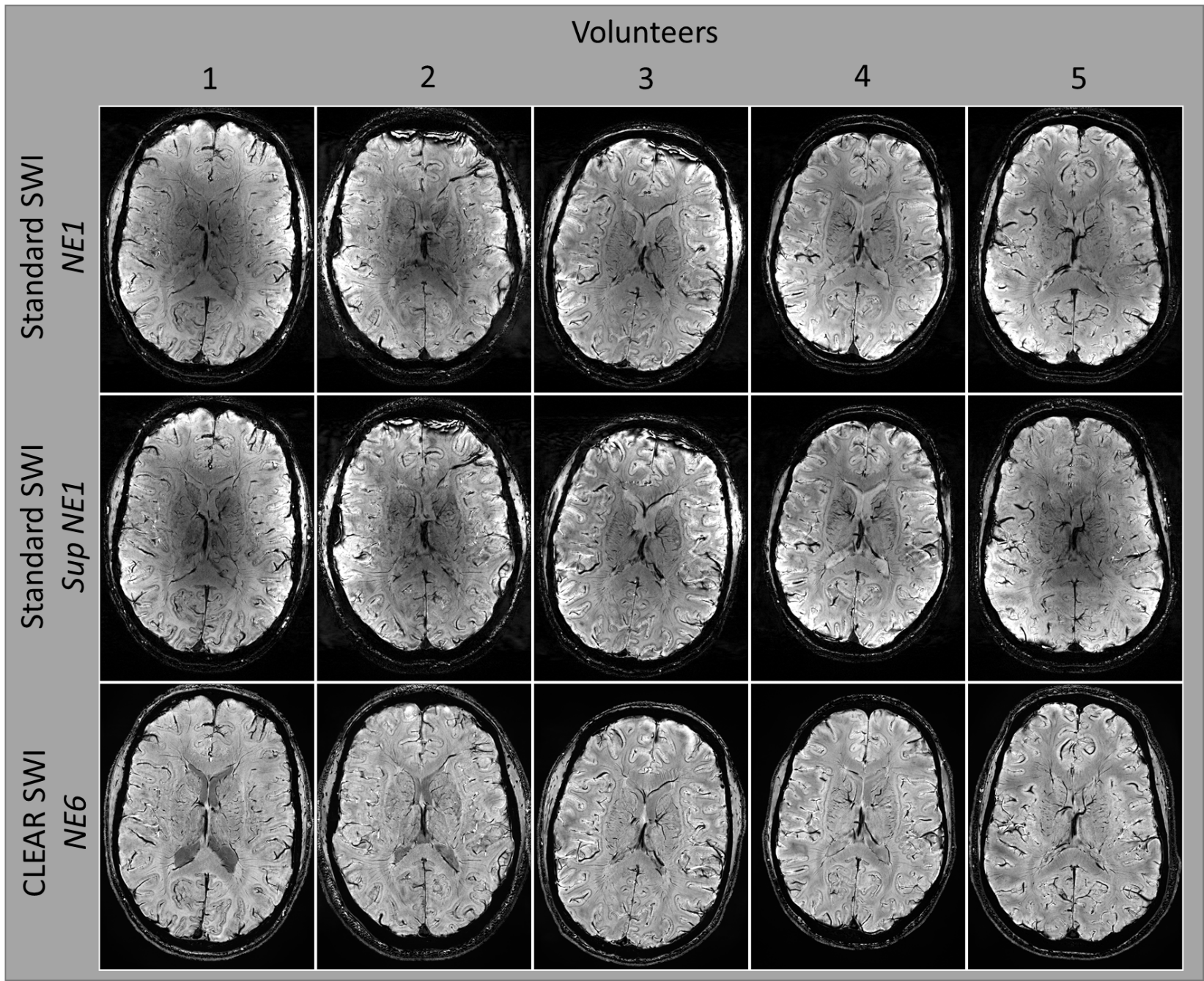


Figure G‑2: Comparison of Standard SWI (bandwidth in top row 60 Hz/px; mid row 100 Hz/px) with CLEAR-SWI (SNR-weighted softplus) in a central slice (circa MNI152 z=+15). The Standard SWIs are inhomogeneous, with hypointensity in the center and patches of hyperintensity close to surface coils. CLEAR-SWI is, in contrast, very homogeneous. Standard SWI for Volunteers 2 and 3 suffers from homodyne artefacts in frontal areas. The internal cerebral veins in Volunteer 2 can be distinguished in CLEAR-SWI, while they appear as one large dark region in both Standard SWI scans.


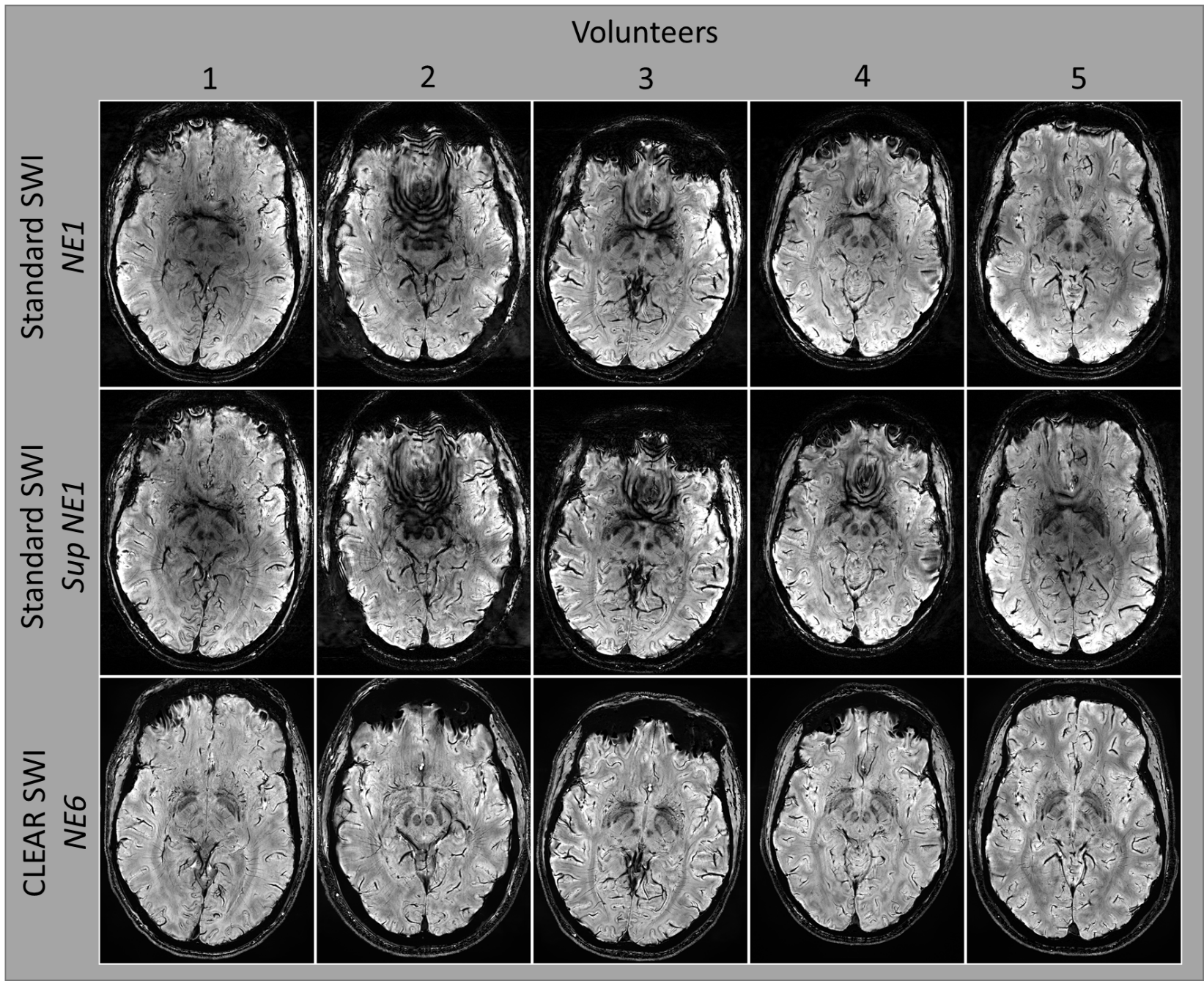


Figure G‑3: Comparison of Standard SWI (bandwidth in top row 60 Hz/px; mid row 100 Hz/px) with CLEAR-SWI (SNR-weighted softplus) in a mid-ventral slice (circa MNI152 z=-7). Standard SWI shows slight (volunteers 1 and 5) to severe (volunteers 2, 3 and 4) homodyne artefacts for all 5 volunteers, while CLEAR-SWI is not affected by wrap-like artefacts and only shows slight phase problems very close to complete signal dropouts. Note that the strong homodyne artefacts seen in volunteers 2, 3 and 4 are artefacts of single SWI slices and not the result of the calculation of minimum intensity projections (mIPs).


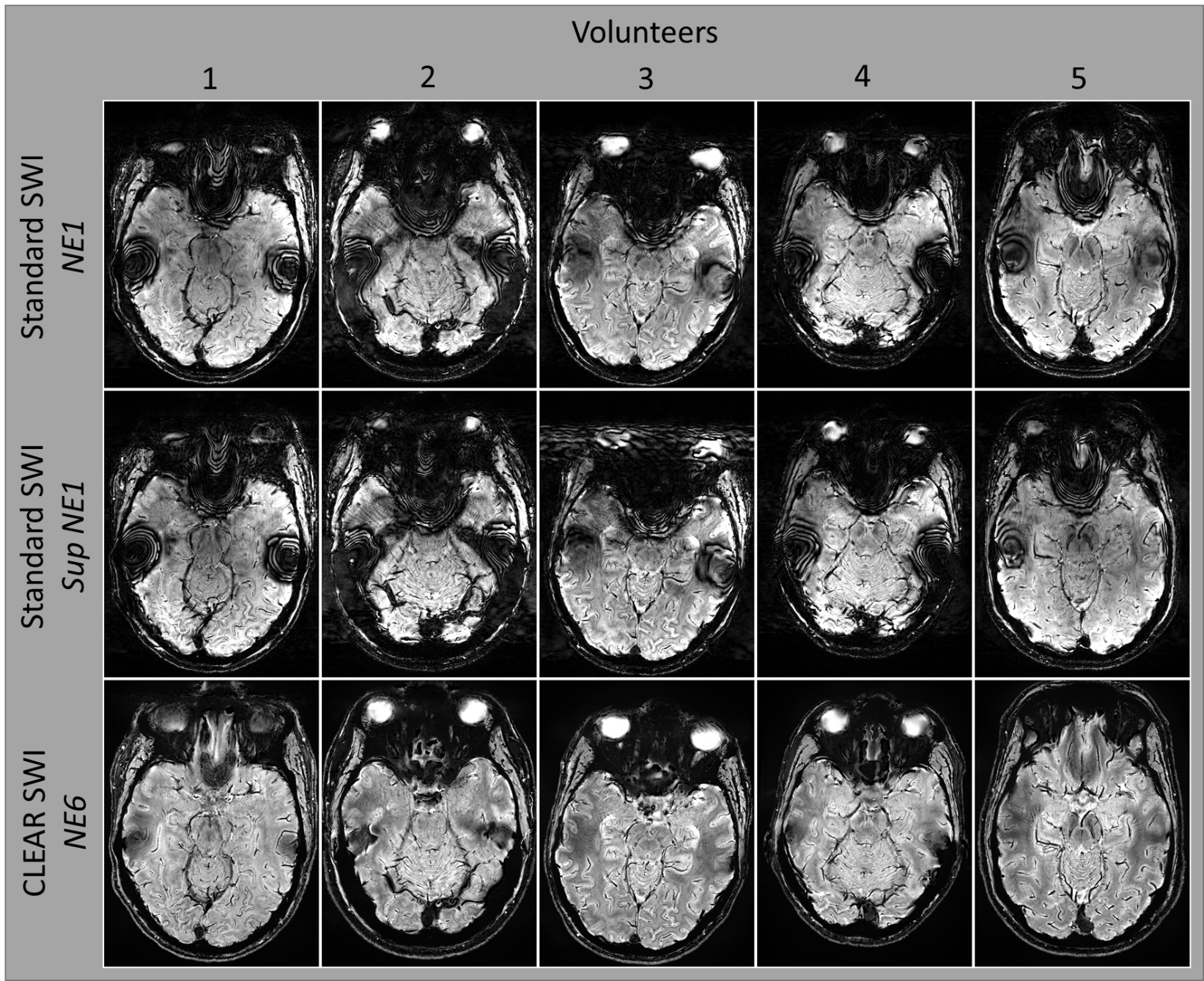


Figure G‑4: Comparison of Standard SWI (bandwidth in top row 60 Hz/px; mid row 100 Hz/px) with CLEAR-SWI (SNR-weighted softplus) in a ventral slice (circa MNI152 z=-15). In all 5 volunteers, CLEAR-SWI retains signal in frontal ventral regions and close to the auditory canals, while considerable parts of the brain have signal dropouts or homodyne artefacts in Standard SWI.

# CLEAR-SWI of all tumor patients from the study

One region has been selected for each of the 13 tumor patients and the CLEAR images (CLEAR-SWI, CLEAR-SWI mIP, CLEAR T_2_^*^-map and CLEAR M_0_) are shown in comparison with Standard SWI and the corresponding mIP. SNR-weighted magnitude combination was used for CLEAR-SWI with softplus scaling. All images were automatically scaled into a linear grayscale range. T_2_^*^ images were scaled to [0;100] ms and all other images were scaled into the [0;1] grayscale range via

Image = Image / quantile(Image, 0.8) * 0.75.

This scaling provided better results for inhomogeneous data (brightening artefact in Standard SWI) than simple rescaling via maximum and minimum into the range [0;1].

**General Findings in 13 Tumor Patients:**

In 10 of the 13 tumor datasets, Standard SWI had homodyne wrap artifacts in the tumor region (see red arrows). In the Standard SWI minimum Intensity Projections (mIPs), these artefacts accumulated over neighboring slices, leading to severe image corruption in several cases. A better visibility of details could be observed in CLEAR-SWI compared to Standard SWI (blue arrows in CLEAR-SWI and CLEAR-SWI mIP). CLEAR-SWI is more homogenous than Standard SWI, which is especially beneficial for mIP (see cases 1,2,5). In many patients, CLEAR T_2_^*^ and CLEAR M_0_ contained information which was not visible in Standard SWI (orange arrows).

Several patients (cases 1,2,4,7,9,13) have visible wrap artefacts in Standard SWI close to the tumor. We do not have biopsy information for these patients, but a likely cause would be high susceptibility values arising from hemorrhage by-products (primarily hemosiderin) leading to strong field changes and homodyne wrap artefacts, what is sometimes referred to as a popcorn lesion appearance in the literature (Mittal et al., 2009).

In general, CLEAR-SWI improved the image quality in the clarity of veins, tumor size and structure and showed reduced artefacts, improved homogeneity and improved visibility of details.


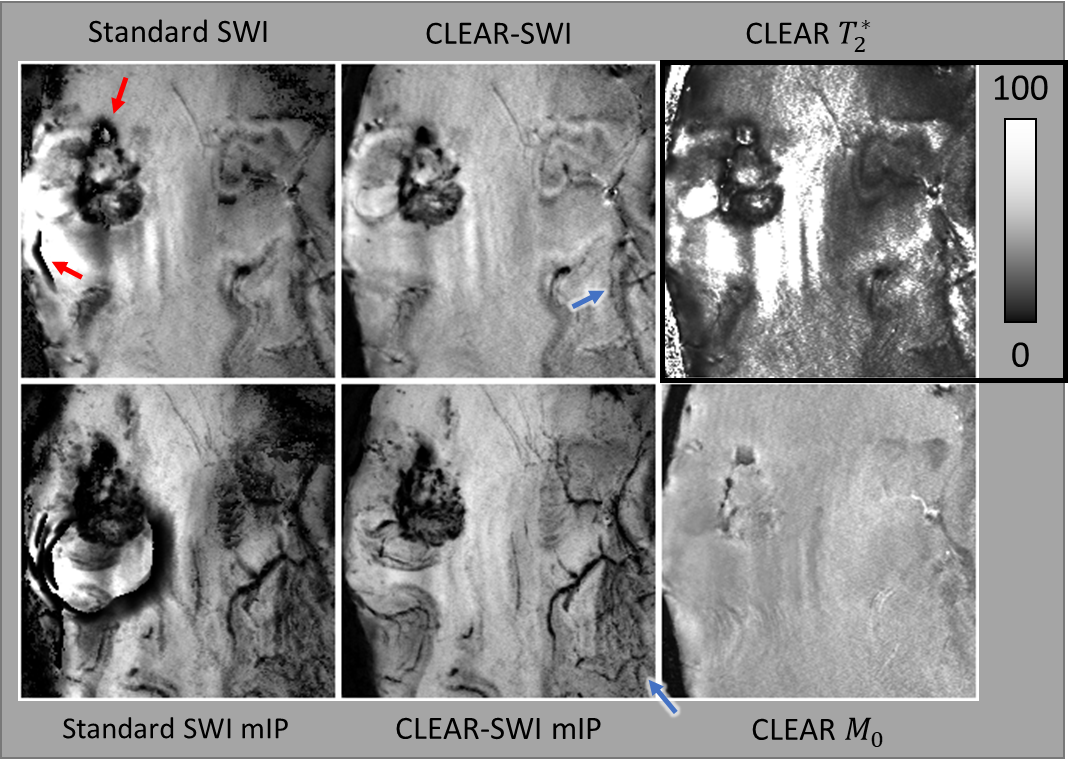


Figure H‑1: Metastasis – Adenocarcinoma; Patient C in Figure 11 – main manuscript.


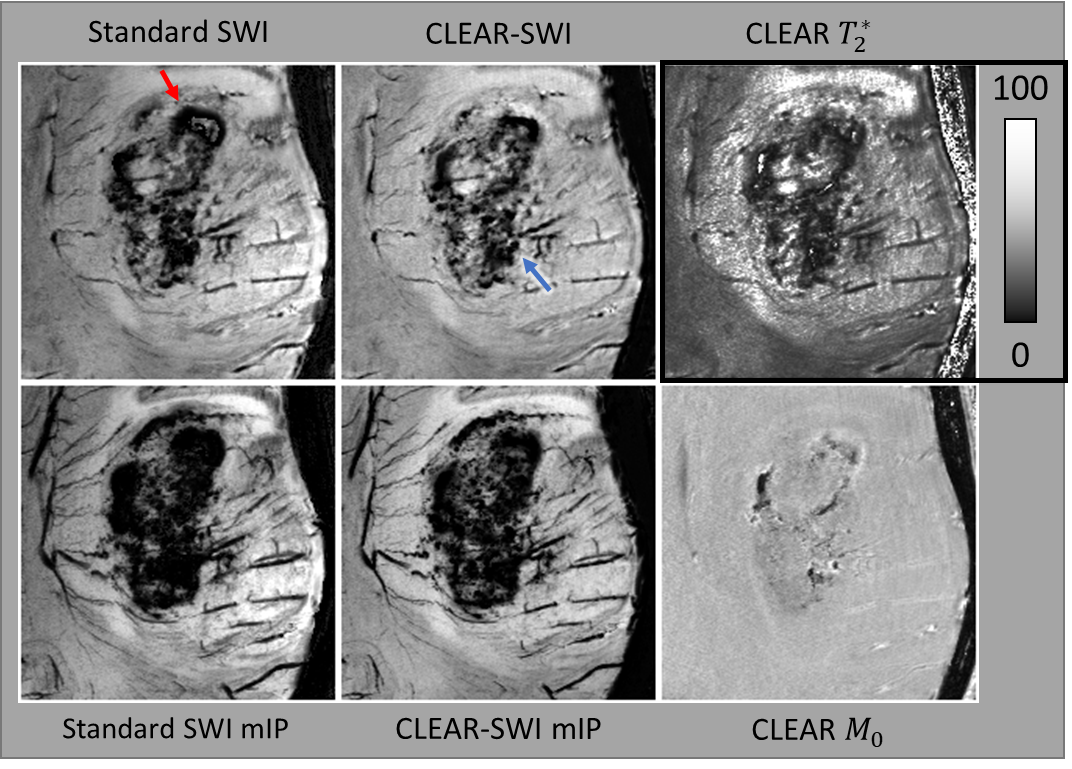


Figure H‑2: Glioblastoma IV


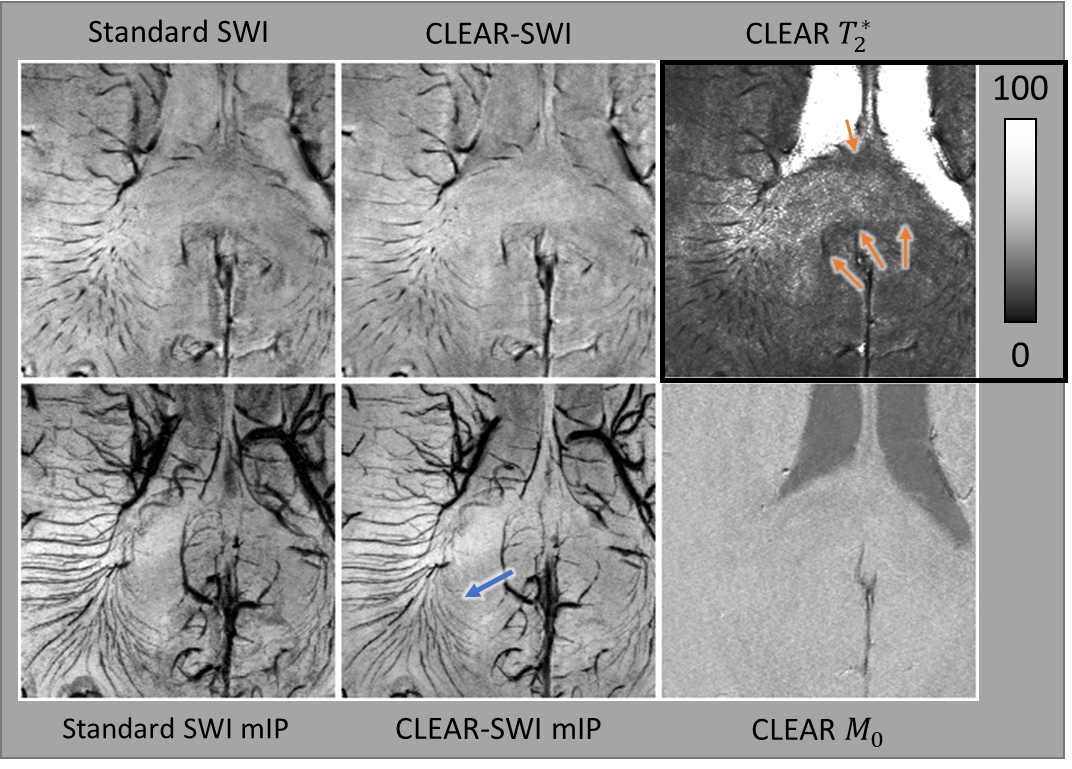


Figure H‑3: Anaplastic Astrocytoma III, with suspected progression to Glioblastoma


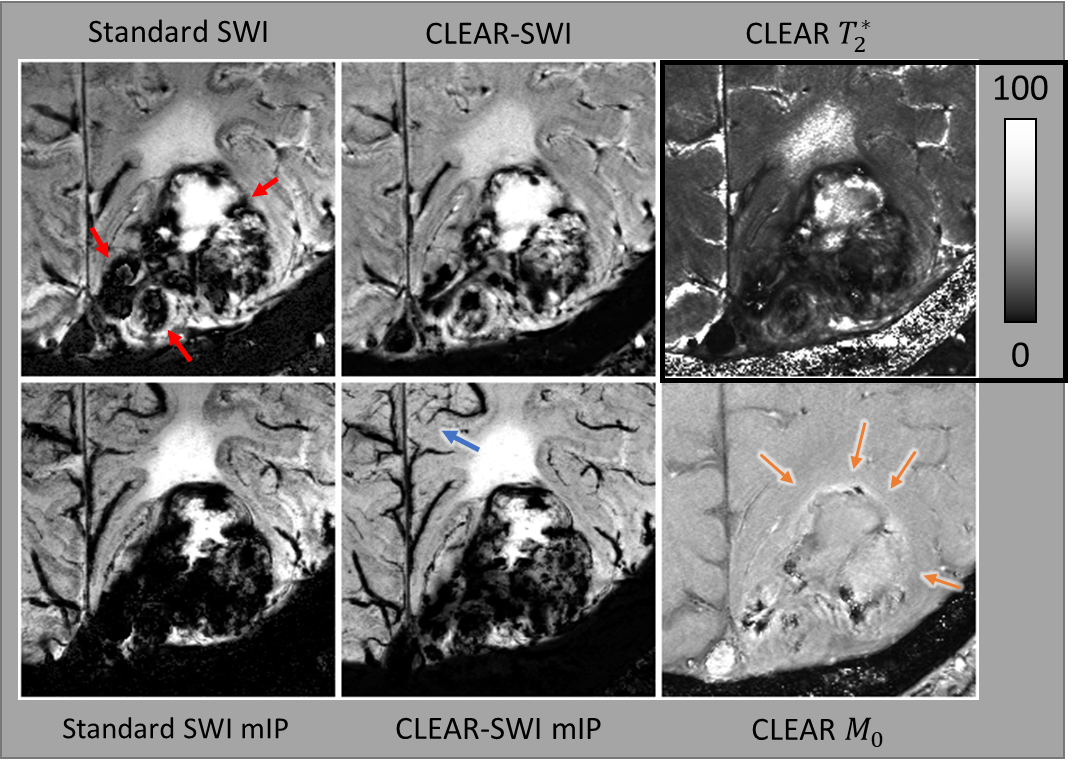


Figure H‑4: Glioblastoma IV and a new lesion


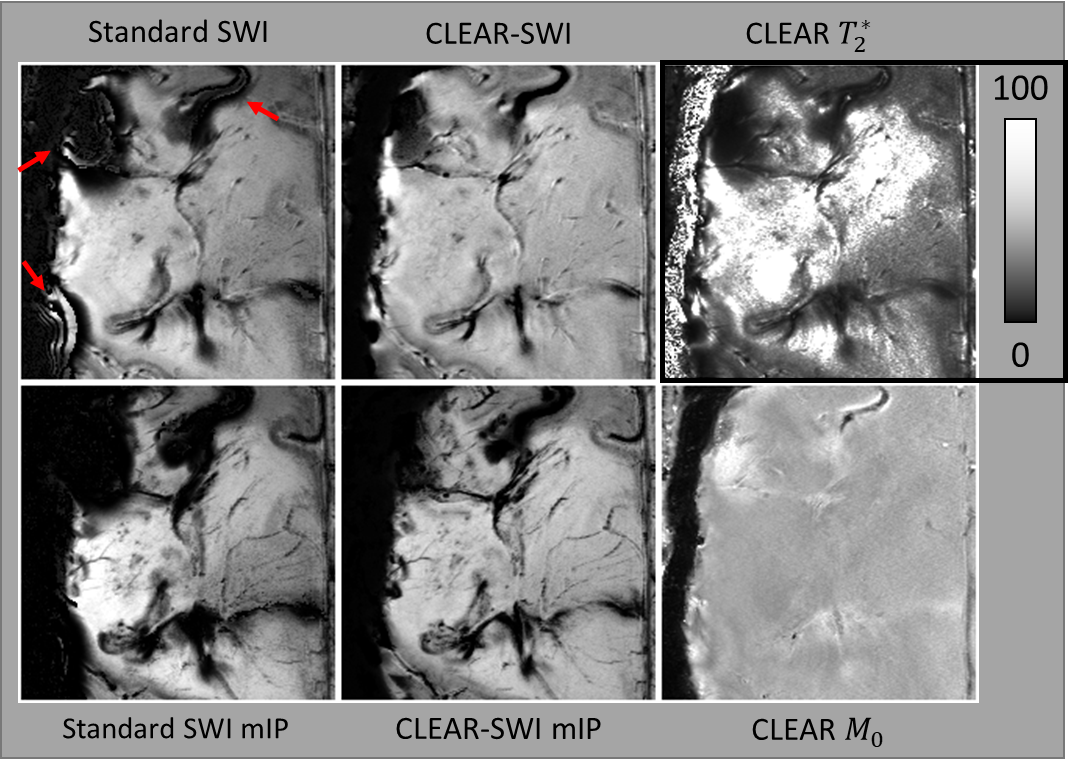


Figure H‑5: Diffuse Astrocytoma II, with suspected progression to Anaplastic Astrocytoma II-III


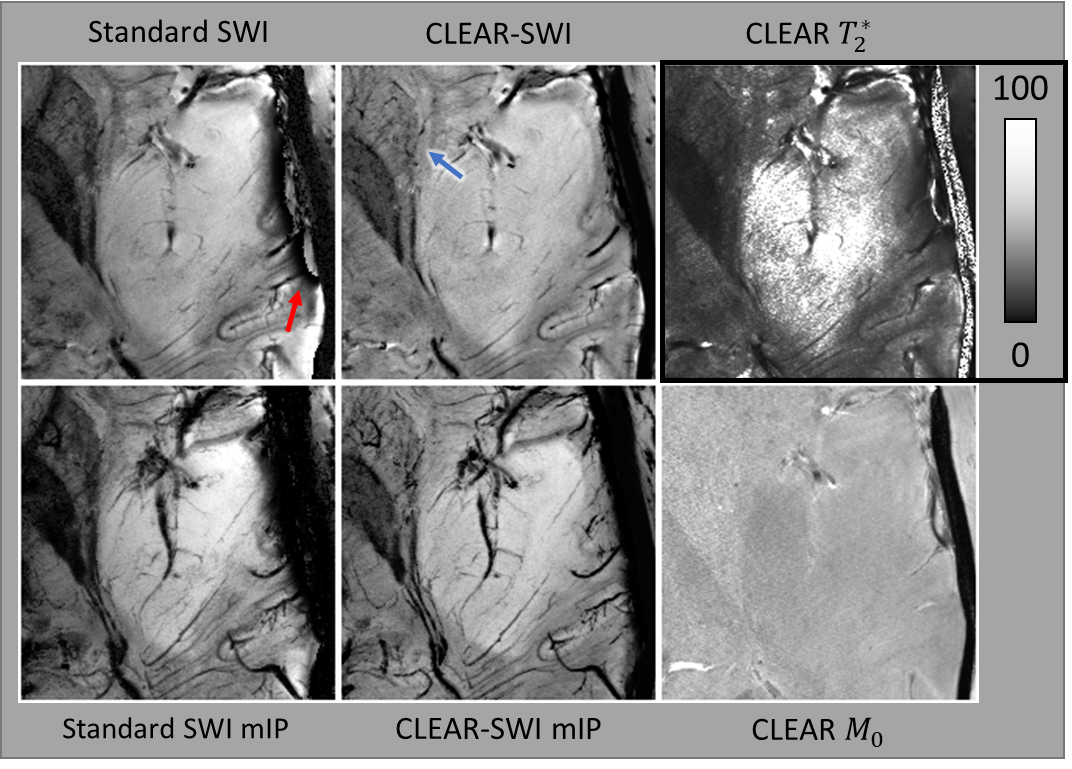


Figure H‑6: Diffuse Astrocytoma II


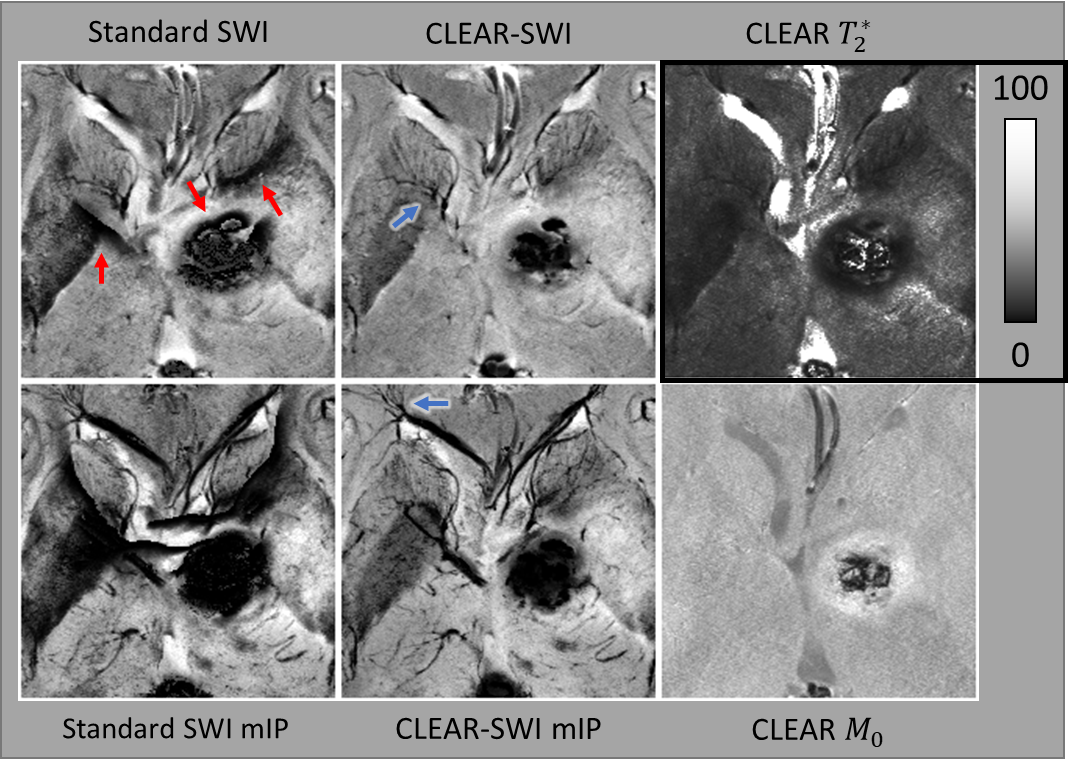


Figure H‑7: Glioblastoma IV; Patient A in Figure 11 – main manuscript.


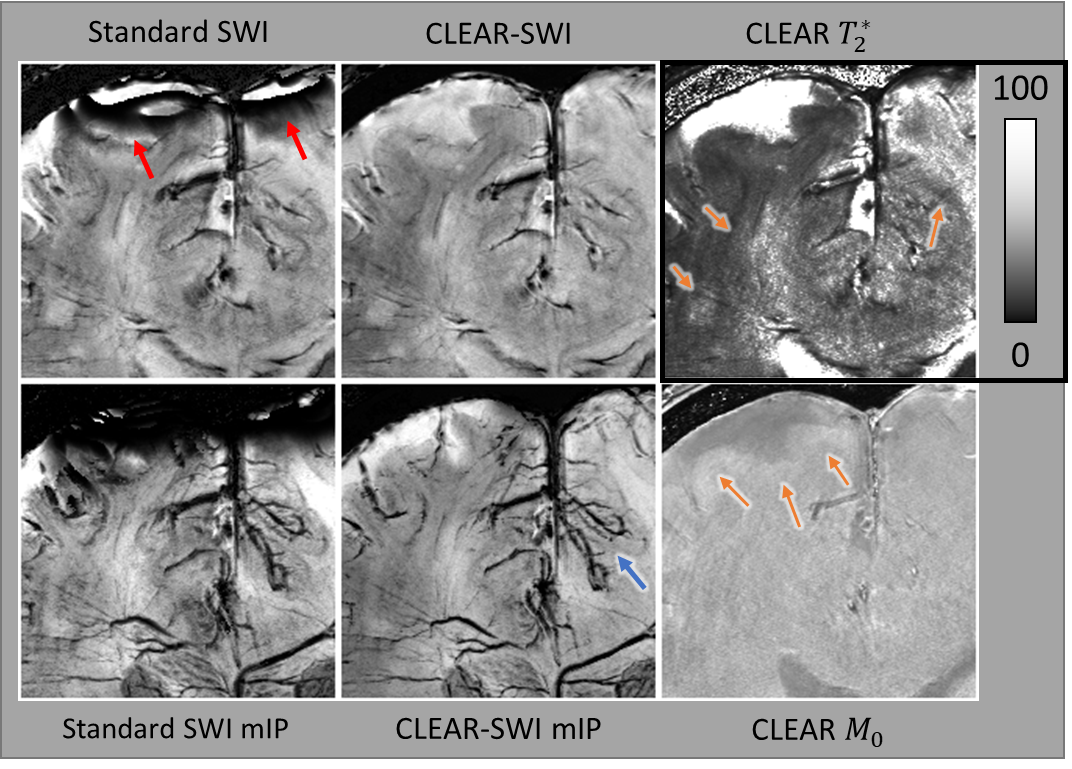


Figure H‑8: Diffuse Astrocytoma II


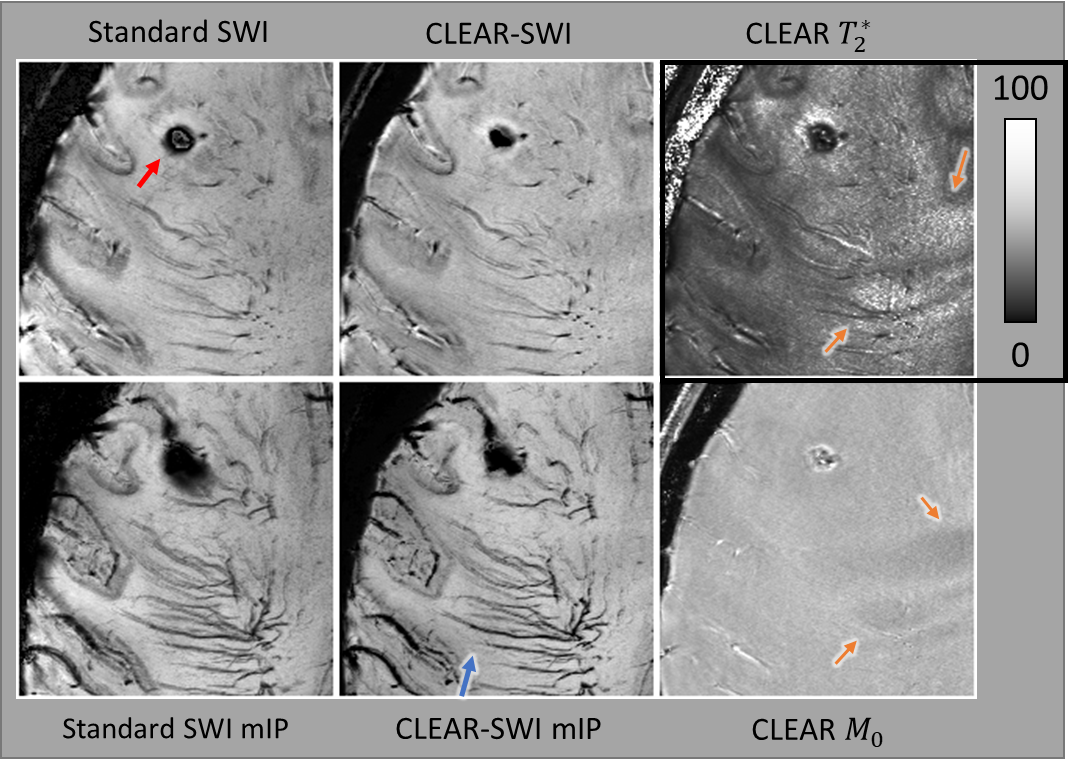


Figure H‑9: Anaplastic Astrocytoma III; Patient B in Figure 11 – main manuscript.


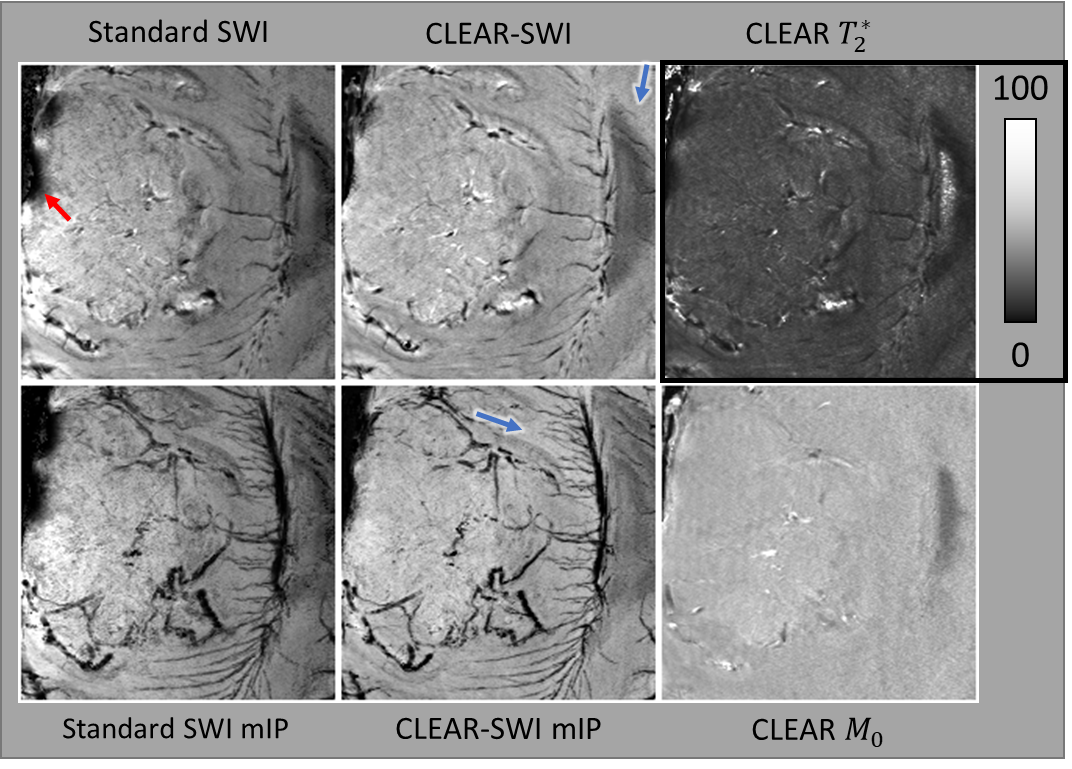


Figure H‑10: Meningothelial Meningioma I


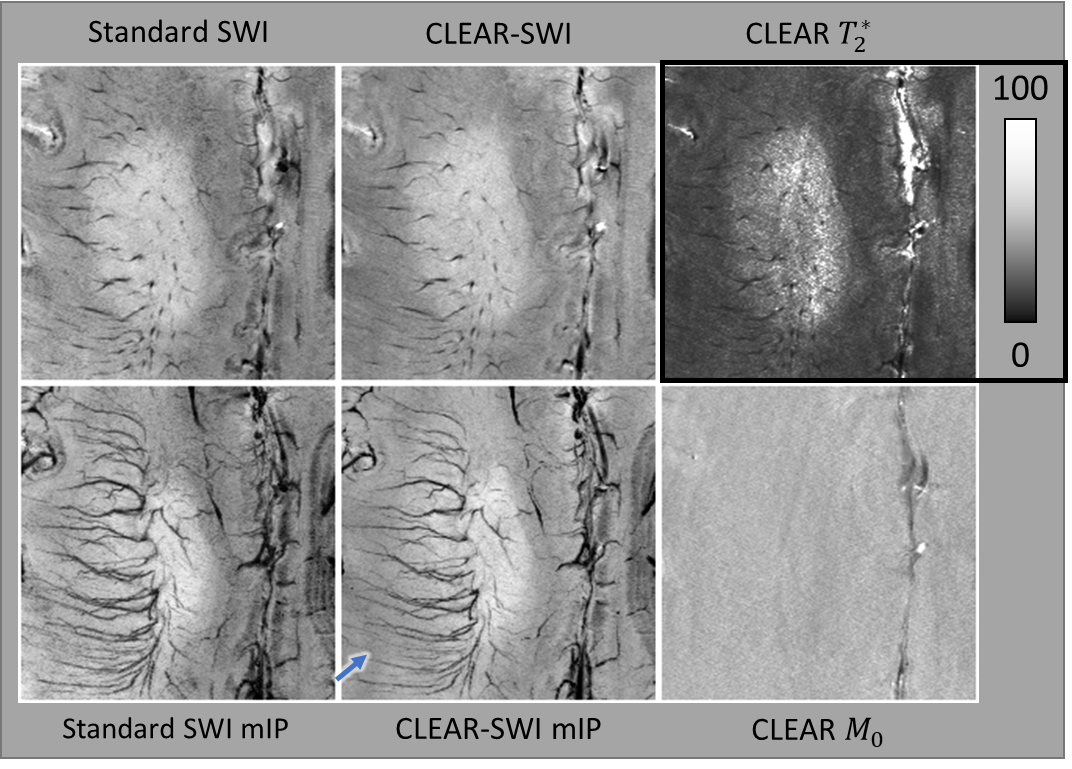


Figure H‑11: Fibroplastic Meningioma I


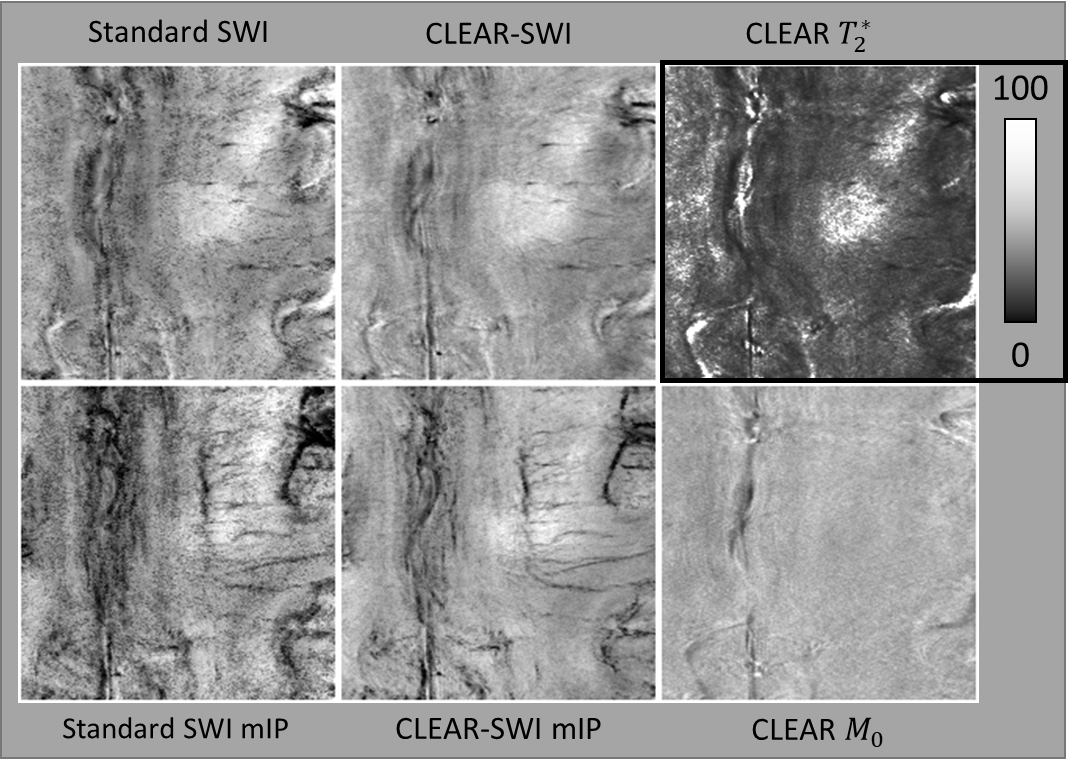


Figure H‑12: Anaplastic Astrocytoma III, with suspected progression to Glioblastoma IV ; Strong motion artefacts


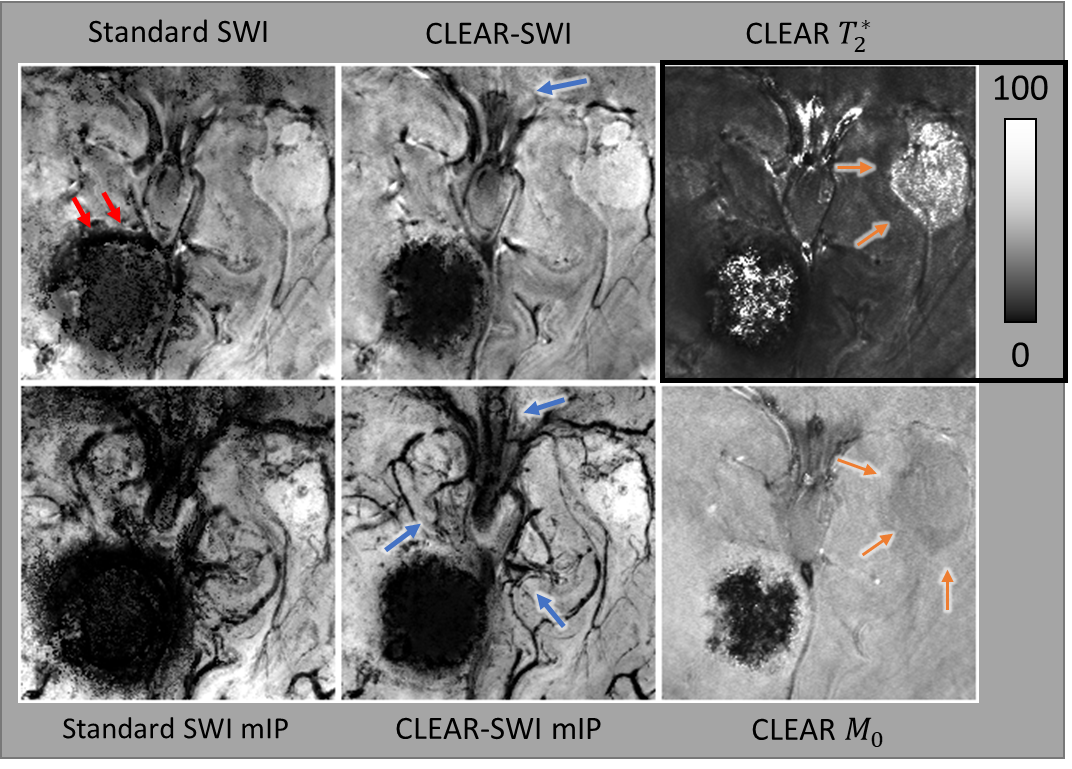


Figure H‑13: Atypical Meningioma II

# References

Luo, J., Jagadeesan, B.D., Cross, A.H., Yablonskiy, D.A., 2012. Gradient Echo Plural Contrast Imaging — Signal model and derived contrasts: T2*, T1, Phase, SWI, T1f, FST2*and T2*-SWI. NeuroImage 60, 1073–1082. https://doi.org/10.1016/j.neuroimage.2012.01.108

Parker, D.L., Payne, A., Todd, N., Hadley, J.R., 2014. Phase reconstruction from multiple coil data using a virtual reference coil. Magn Reson Med 72, 563–569. https://doi.org/10.1002/mrm.24932

Quinn, M.P., Gati, J.S., Klassen, L.M., Lin, A.W., Bird, J.R., Leung, S.E., Menon, R.S., 2014. Comparison of Multiecho Postprocessing Schemes for SWI with Use of Linear and Nonlinear Mask Functions. AJNR Am J Neuroradiol 35, 38–44. https://doi.org/10.3174/ajnr.A3584

Robinson, S.D., Bredies, K., Khabipova, D., Dymerska, B., Marques, J.P., Schweser, F., 2017a. An illustrated comparison of processing methods for MR phase imaging and QSM: combining array coil signals and phase unwrapping. NMR in biomedicine 30. https://doi.org/10.1002/nbm.3601

Robinson, S.D., Dymerska, B., Bogner, W., Barth, M., Zaric, O., Goluch, S., Grabner, G., Deligianni, X., Bieri, O., Trattnig, S., 2017b. Combining phase images from array coils using a short echo time reference scan (COMPOSER). Magnetic Resonance in Medicine 77, 318–327. https://doi.org/10.1002/mrm.26093

1. https://github.com/korbinian90/CLEARSWI.jl [↑](#footnote-ref-2)
